# Rational design and computational evaluation of a multi-epitope vaccine for monkeypox virus: Insights into binding stability and immunological memory

**DOI:** 10.1016/j.heliyon.2024.e36154

**Published:** 2024-08-20

**Authors:** Anupamjeet Kaur, Amit Kumar, Geetika Kumari, Rasmiranjan Muduli, Mayami Das, Rakesh Kundu, Suprabhat Mukherjee, Tanmay Majumdar

**Affiliations:** a National Institute of Immunology, New Delhi, India; b Department of Zoology, Visva-Bharati University, Santiniketan, West Bengal, India; c Department of Animal Science, Kazi Nazrul University, Asansol, West Bengal, India

**Keywords:** Immunoinformatics, Multi-epitope hybrid vaccine, Monkeypox virus, MD simulation, Immune simulation

## Abstract

Multi-epitope vaccines strategically tackle rapidly mutating viruses by targeting diverse epitopes from different proteins, providing a comprehensive and adaptable immune protection approach for enhanced coverage against various viral variants. This research employs a comprehensive approach that includes the mapping of immune cells activating epitopes derived from the six structural glycoproteins (A29L, A30L, A35R, L1R, M1R, and E8L) of Monkeypox virus (Mpox). A total of 7 T-cells-specific epitopes, 13 B-cells-specific epitopes, and 5 IFN-γ activating epitopes were forecasted within these glycoproteins. The selection process focused on epitopes indicating high immunogenicity and favorable binding affinity with multiple MHC alleles. Following this, a vaccine has been formulated by incorporating the chosen epitopes, alongside adjuvants (PADRE peptide) and various linkers (EAAAK, GPGPG, and AAY). The physicochemical properties and 3D structure of the multi-epitope hybrid vaccine were analysed for characterization. MD simulations were employed to predict the binding stability between the vaccine and various pathogen recognition receptors such as TLRs (TLR1, TLR2, TLR4, and TLR6), as well as both class I and II MHC, achieved through hydrogen bonding and hydrophobic interactions. Through *in silico* cloning and immune simulation, it was observed that the multi-epitopes vaccine induced a robust memory immune response upon booster doses, forecasting protective immunity upon viral challenge. This protective immunity was characterized by the production of IgM + IgG antibodies, along with release of inflammatory cytokines like IFN-γ, and IL12, and the activation of various immune cells. This study offers valuable insights into the potential of a multi-epitope vaccine targeting the Mpox virus.

## Highlights

• Six glycoproteins from the Mpox virus were identified and evaluated to construct a multi-epitope hybrid vaccine.
• The vaccine demonstrated strong antigenicity, immunogenicity, and was assembled with specific epitopes, adjuvant, and linkers.
• Physicochemical properties, 3D structure, and stability were analyzed; docking simulations confirmed immune receptor interactions.
• Immune simulations revealed a robust response, with antibody production, immune cell activation, and enhanced memory post-booster.
• The study underscores immunoinformatics' potential in developing a promising multi-epitope Mpox vaccine.

## Graphical abstract

A comprehensive strategy for designing a vaccine against the Monkeypox virus was implemented, utilizing various multi-epitopes from six structural glycoproteins that activate immune cells to generate protective immune-memory response against virus challenge.

**Figure:**
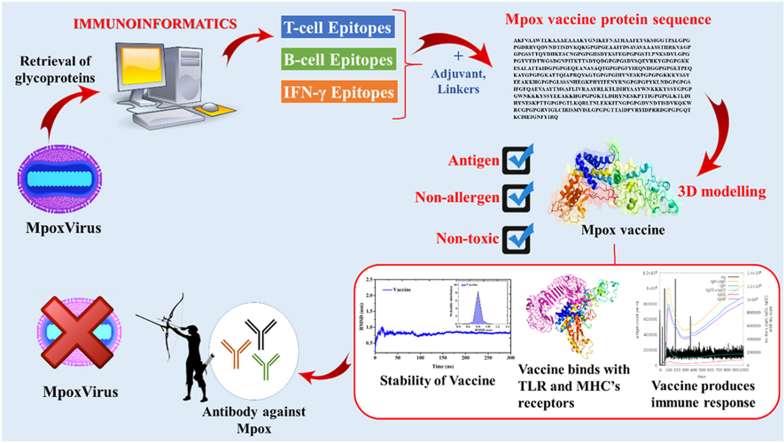

## Importance

Amid the escalating global menace posed by the Mpox, this study assumes paramount importance. Historically, Mpox has been underserved in terms of attention and research funding. With now categorizes Mpox as an "evolving threat of moderate public health concern," underscoring the pressing demand for vaccine development. Multi-epitope vaccines elicit a robust, mutation-resistant immune response by incorporating diverse viral protein epitopes. This complexity minimizes the emergence of escape mutants, as simultaneous mutations in multiple epitopes are less likely. Cross-reactive immune responses offer sustained protection against significant mutations in one protein. Concurrent targeting of multiple epitopes applies mutational pressure, challenging the virus to evade the immune response. Utilizing immunoinformatics, creating a Mpox vaccine is crucial. *In silico* simulations validate its efficacy, predicting robust and enduring immune responses. This study introduces a proactive strategy for heightened protection against Mpox and other emerging infectious diseases, extending beyond the immediate outbreak response.

## 1 Introduction

Monkeypox (Mpox) virus is a member of the Orthopoxvirus genus and belongs to the family Poxviridae [1]. The Mpox virus, initially detected in monkeys at a Danish laboratory in 1958, made its first appearance in humans in 1970, when a nine-month-old baby in the Democratic Republic of the Congo (DRC) contracted the virus [2]. This virus predominantly circulates in Central and West African nations, specifically in remote, forested regions. While sporadic outbreaks occur in these areas, isolated cases have been recorded outside of Africa, with reports in both the United States and the United Kingdom. Despite historical prevalence in endemic regions, the Mpox virus has recently surged, with over 3000 documented cases in 50+ countries since May 2022. Classified by the WHO as an "evolving threat of moderate public health concern" since June 23, 2022 [3,4], Mpox is a 197 kb double-stranded DNA virus with 197 non-overlapping ORFs. While primarily affecting animals, it can infect humans. A recent study discussed the first known case of someone having Monkeypox virus, COVID-19 (caused by SARS-CoV-2), and HIV-1 all at once. The patient showed symptoms of both Monkeypox and COVID-19, showing that these infections can have similar symptoms. This case highlights the need to think about co-infections in people who have traveled to places with Monkeypox outbreaks and to use the right tests for those at higher risk [5]. Men living with HIV are more likely to be affected by monkeypox, with compromised immune systems leading to severe disease. More research is needed on treatments targeting the immunopathology of monkeypox infection, as well as the potential for antibody-dependent enhancement [6]. Li et al., have studied that monkeypox is mainly transmitted through sexual contact, particularly among men who have sex with men (MSM). This represents a new route of transmission for monkeypox virus (MPXV) and highlights the importance of considering sexual transmission in the current outbreak. The B.1 lineage of MPXV, closely related to the current outbreak, has undergone microevolution and formed several clusters, indicating ongoing viral evolution [7].

The U.S. Mpox vaccination plan offers ACAM2000 [8] and JYNNEOS for individuals aged 18 and older. JYNNEOS, licensed in 2019, is designed for fewer side-effects but exhibits limited B-cell activation compared to ACAM2000. In contrast, monkeypox virus infection induces robust B-cell and T-cell responses [9]. T-helper (Th) cells activate B cells, crucial for the immune response, while T cytotoxic (Tc) cells eliminate infected cells. Memory response involves T (Tmemory) and B-cells (Bmemory) "remembering" pathogens for a quicker response. B-cells differentiate into plasma cells, producing antibodies to neutralize pathogens. Collaboration among Th, Tc and B-cells enhances the adaptive immune system for lasting defense. Germinal center activation refines responses; in lymph nodes, B-cells undergo maturation and class-switching, aided by Th cells and follicular dendritic cells. This process selects high-affinity B-cells, producing antibodies with enhanced capabilities, contributing to long-lasting immune memory against recurrent infections. To obtain T-memory and B-memory response against multiple epitopes of various protein, we have opted for a reverse vaccinology-based immunoinformatics strategy for crafting multi-epitope hybrid vaccines. Pathogen recognition receptors (PRRs), such as Toll-like receptors (TLRs), play a vital role in initiating the protective immune response and establishing immunological memory, contributing to the effectiveness of vaccination.

The Monkeypox (Mpox) virus contains critical glycoproteins crucial for its lifecycle and pathogenicity, chosen for their pivotal roles in the immune response against Mpox infections. The aim of this study is to design and evaluate a multi-epitope vaccine for the Mpox virus by targeting diverse epitopes from six structural glycoproteins (A29L, A30L, A35R, L1R, M1R, and E8L) of the Mpox virus to provide comprehensive immune protection. Among these glycoproteins, A29L facilitates virus entry, A30L aids in viral attachment, A35R contributes to immune evasion, L1R is essential for virus assembly and release, M1R promotes virus assembly, and E8L modulates host immune responses, facilitating viral replication [10]. The hypothesis is that incorporating highly immunogenic epitopes (T-cell, B-cell as well as IFN-γ) with favorable MHC binding, along with adjuvants and linkers, will induce a robust memory immune response, demonstrated through *in silico* analyses, and predict protective immunity upon viral challenge. Through the application of diverse parameters, including *in silico* immune simulation and molecular dynamics (MD) simulation, we envision that these multi-epitope vaccines offer a strategic advantage in addressing the challenges posed by rapidly mutating viruses. By focusing on multiple epitopes across diverse proteins, these vaccines represent a more comprehensive and adaptable approach to immune protection, potentially broadening coverage against a varied spectrum of viral variants.

## 2 Results

### 2.1 Retrieval and characterization of protein sequences

In this study, we have focused on six glycoproteins (A29L, A30L, A35R, L1R, M1R, and E8L) of Mpox (strain: Singapore 2019) obtained from NCBI, aiming to develop a multi-epitope hybrid vaccine against the Mpox virus. Firstly, we assessed the antigenicity and allergenicity of all glycoprotein sequences (Table 1). Among the examined glycoproteins, A30L, A35R, M1R, and E8L proteins demonstrated high antigenicity scores, surpassing 0.4. As anticipated, all these glycoproteins, except for L1R, were classified as non-allergenic. Subsequently, we proceeded to analyze the physiochemical properties of these glycoproteins (Table 2). The glycoproteins showed a predicted isoelectric point (pI) ranging from 5.16 to 7.77, indicating that A29L, A30L, A35R, L1R, and M1R proteins are weakly acidic, while E8L is neutral in nature. Furthermore, among these viral glycoproteins, A29L, A30L, L1R, and M1R exhibited an instability index below 40, suggesting their inherent stability. The other physiochemical parameters such as size, molecular weight, aliphatic index, and GRAVY are provided in Table 2. Moreover, we conducted predictions for the glycoprotein's secondary conformation, which unveiled diverse proportions of α-helix, extended strand, and random coil elements (Table 3).

**Table 1:** Antigenicity score and allergenicity score of target structural glycoproteins.

Structural Glycoprotein	Antigenicity Score	Antigenicity	Allergenicity
A29L	0.3277	NON-ANTIGEN	PROBABLE NON-ALLERGEN
A30L	0.6212	ANTIGEN	PROBABLE NON-ALLERGEN
A35R	0.4998	ANTIGEN	PROBABLE NON-ALLERGEN
L1R	0.3459	NON-ANTIGEN.	PROBABLE ALLERGEN
M1R	0.6339	ANTIGEN	PROBABLE NON-ALLERGEN
E8L	0.5316	ANTIGEN	PROBABLE NON-ALLERGEN

**Table 2:** Physiochemical properties of structural glycoproteins.

Structural Glycoprotein	Size	Molecular weight	Theoretical pI	Instability Index	Aliphatic Index	GRAVY
A29L	110	12559.24	5.73	32.28	73.73	−0.75
A30L	146	16403.68	6.54	29.97	86.78	0.057
A35R	181	20023.43	5.16	41.03	72.76	−0.316
L1R	152	17795.39	5.39	33.33	91.71	−0.257
M1R	250	27303.24	6.72	33.47	87.48	−0.004
E8L	304	35247.99	7.77	45.45	88.22	−0.359

**Table 3:** Secondary structural components of target structural glycoproteins.

Structural glycoprotein	α- helix%	3_10_ helix%	π-helix%	β bridge%	Extended Strand%	β turn%	β region%	Random coil%	ambiguous state%	other states%
A29L	62	0	0	0	6	1	0	31	0	0
A30L	43	0	0	0	25	8	0	24	0	0
A35R	39	0	0	0	18	3	0	40	0	0
L1R	54	0	0	0	11	5	0	30	0	0
M1R	52	0	0	0	19	2	0	28	0	0
E8L	28	0	0	0	21	7	0	44	0	0

During our study, we thoroughly assessed the immunogenicity, immune response, allergic potential, and physical-chemical attributes of each epitope. Subsequently, we selectively chose epitopes with high immogenicity and lacks allergenicity for the hybrid vaccine.

### 2.2 Prediction of T-cells, B-cells and IFN-γ specific epitopes

Integrating Tc-cells specific epitopes into vaccine design is crucial, as it plays a fundamental role in triggering a robust cellular immune response. These epitopes are specifically designed to target virus-infected cells, offering cross-protection, establishing long-term memory response, and effectively combating viral infections [11]. We have identified epitopes activating Tc-cells in all six glycoproteins, consisting 4 epitopes in A29L, 11 epitopes each in A30L, A35R, and L1R, 16 epitopes in M1R, and 21 epitopes in E8L proteins (Table 4). The identified Tc-cells specific epitopes demonstrate a strong binding affinity to multiple MHC class I alleles and their supertypes. Similarly, the significance of Th cells specific epitopes is pivotal in promoting the differentiation of follicular T-cell subsets (TFHs) which eventually develops T-memory responses. These epitopes also play a key role in activating B-cells either directly or through activation of TFHs and regulating the production of antibodies from plasma cells. During our investigation, we identified 8 Th-epitopes in the A29L protein, 14 in A35R, 2 in L1R, 5 in M1R, and 19 in E8L protein. Notably, all of these epitopes displayed robust binding affinity to multiple alleles of MHC class II (Table 5). Both Th and Tc specific epitopes have demonstrated high immunogenicity, characterized by favorable antigenicity scores and non-allergenic properties. In addition, using the IEDB tool, we predicted continuous B-cell epitopes based on factors such as hydrophilicity, exposed surface, polarity, and antigenic propensity. Thirteen B-cell epitopes were carefully chosen for their antigenicity and lacking allergenicity. These epitopes comprise 1 in A30L, 4 in A35R, 1 in L1R, 4 in M1R, and 3 in E8L protein (Table 6). Likewise, we conducted predictions for discontinuous epitopes for B-cell located on the surface of all glycoproteins, and the relevant data is presented in Table 7.

**Table 4:** The overlapped CTL epitopes in Mpox virus glycoproteins.

CTL	Supertypes/HLA Alleles	Antigenicity score (>4.0)	Allergenicity
**A29L**			
^101^DVQTGRHPY^109^	A1, A26	1.3329	NON-ALLERGEN
^48^KQRLTNLEK^56^	A3, B27	0.6546	NON-ALLERGEN
^46^TLKQRLTNL^54^	HLA-A*30:01, HLA-B*08:01	0.9942	NON-ALLERGEN
^88^TLRAAMISL^96^	A2, B7, HLA-A*02:03, HLA-A*03:01	1.1323	NON-ALLERGEN
**A30L**			
^12^ATAAVCLLF^20^	A1, A24, A26, B58, B62	0.6308	NON-ALLERGEN
^38^ATHAAFEYSK^47^	A24, A26, B8, B62	0.8034	NON-ALLERGEN
^35^EFNATHAAF^43^	A2, B8	1.5598	NON-ALLERGEN
^7^FFIVVATAAV^16^	A26, B58, B62	0.7002	NON-ALLERGEN
^8^FIVVATAAV^16^	A3, B27, B62, B39, B58, B62	0.6997	NON-ALLERGEN
^115^FTFSDVINI^123^	A1, B58	1.0007	NON-ALLERGEN
^34^KEFNATHAAF^43^	HLA-A*68:02, HLA-A*68:02, HLA-A*01:01	1.2406	NON-ALLERGEN
^18^LLFIQSYSI^26^	HLA-A*02:06, HLA-A*32:01, HLA-A*02:01, HLA-A*02:01	0.4123	NON-ALLERGEN
^2^NSLSIFFIV^10^	HLA-A*02:03, HLA-A*23:01, HLA-B*35:01, HLA-A*02:06	0.5713	NON-ALLERGEN
^87^SIFGFQAEV^95^	HLA-A*23:01, HLA-A*02:01, HLA-A*01:01, HLA-B*44:03, HLA-A*30:02, HLA-A*68:02	0.4227	NON-ALLERGEN
^3^SLSIFFIVV^11^	HLA-A*02:03, HLA-B*35:01	0.8068	NON-ALLERGEN
**A35R**			
^37^IRISMVISL^45^	A1, A3, A26, B58, B62	1.3141	NON-ALLERGEN
^49^ITMSAFLIV^57^	A1, B58, B62	0.6910	NON-ALLERGEN
^97^KESCNGLYY^105^	A2, A24, A26, B62, A2, B62	0.5017	NON-ALLERGEN
^46^LSMITMSAF^54^	B58, B62, A24, B39	0.7548	NON-ALLERGEN
^46^LSMITMSAFL^55^	HLA-A*02:01, HLA-A*68:02	0.5394	NON-ALLERGEN
^48^MITMSAFLI^56^	HLA-A*02:06, HLA-A*02:03	0.4325	NON-ALLERGEN
^48^MITMSAFLIV^57^	HLA-A*23:01, HLA-A*68:02, HLA-A*68:01, HLA-A*02:03	0.5362	NON-ALLERGEN
^41^MVISLLSMI^49^	HLA-A*02:06, HLA-A*02:03, HLA-A*68:01, HLA-A*11:01, HLA-A*02:03	0.5383	NON-ALLERGEN
^47^SMITMSAFLI^56^	HLA-A*30:02, HLA-A*68:01, HLA-A*02:06, HLA-A*68:01	0.4361	NON-ALLERGEN
^40^SMVISLLSM^48^	HLA-B*15:01, HLA-A*02:01, HLA-A*02:06, HLA-B*15:01	0.7405	NON-ALLERGEN
^50^TMSAFLIVR^58^	HLA-A*11:01, HLA-A*02:01	0.577	NON-ALLERGEN
**L1R**			
^48^ALATTAIDPV^57^	HLA-A*02:01, HLA-A*02:03, HLA-A*02:01	1.0757	NON-ALLERGEN
^36^FVISLMRFK^44^	A3, A26, HLA-A*02:03, HLA-A*02:06	0.7848	NON-ALLERGEN
^36^FVISLMRFKK^45^	HLA-B*15:01, HLA-B*08:01	0.5201	NON-ALLERGEN
^31^GYLFDFVISL^40^	HLA-A*11:01, HLA-A*30:01	0.4699	NON-ALLERGEN
^39^QYLDFLLLLL^48^	HLA-A*68:01,HLA-A*02:01	1.2543	NON-ALLERGEN
^38^TQYLDFLLL^46^	B39, B44, B62	1.2541	NON-ALLERGEN
^38^TQYLDFLLLL^47^	HLA-B*08:01, HLA-A*68:01	1.0657	NON-ALLERGEN
^37^VISLMRFKK^45^	HLA-A*02:06, HLA-A*68:02	0.5838	NON-ALLERGEN
^40^YLDFLLLLL^48^	A1, A2, B39, HLA-A*23:01, HLA-A*68:01	1.195	NON-ALLERGEN
^32^YLFDFVISL^40^	A2, A26, B39, B62, HLA-A*02:06, HLA-B*15:01,HLA-A*30:02	0.7273	NON-ALLERGEN
^32^YLFDFVISLM^41^	HLA-B*15:01. HLA-A*03:01, HLA-A*02:06	0.6365	NON-ALLERGEN
**M1R**			
^57^AALFMYYAK^65^	A3, A26, B58, B62	0.6427	NON-ALLERGEN
^57^AALFMYYAKR^66^	A1, A3, A26, B58, B62	0.7442	NON-ALLERGEN
^19^AMFTAALNI^27^	A24, B8	0.3357	NON-ALLERGEN
^23^DTFFRTSPM^31^	A26, B58, B62	0.0517	NON-ALLERGEN
^60^FMYYAKRML^68^	A26, B62	0.4498	NON-ALLERGEN
^60^FMYYAKRMLF^69^	A1, B62	0.5830	NON-ALLERGEN
^54^IILAALFMY^62^	A1, B58, B62	0.2423	NON-ALLERGEN
^54^IILAALFMYY^63^	B7, B8, B39, A1, A24, A26, B58, A2, A24, B8, B62	0.3527	NON-ALLERGEN
^55^ILAALFMYYA^64^	HLA-A*68:01, HLA-B*15:01	0.2906	NON-ALLERGEN
^49^IVIGVIILA^57^	HLA-B*58:01, HLA-A*68:01	0.8296	NON-ALLERGEN
^5^KIKLILANK^13^	HLA-A*02:01, HLA-A*31:01	0.7665	NON-ALLERGEN
^196^LAALFMYYAK^205^	HLA-A*68:02, HLA-A*11:01, HLA-A*02:03,HLA-B*15:01	0.6688	NON-ALLERGEN
^220^LANKENVHW^228^	HLA-B*07:02, HLA-A*68:01, HLA-A*68:01, HLA-A*30:01	1.7164	NON-ALLERGEN
^13^TLSERISSK^21^	HLA-A*30:01,HLA-B*57:01	0.7769	NON-ALLERGEN
^9^TTVNTLSER^17^	HLA-A*26:01,HLA-A*24:02	0.4993	NON-ALLERGEN
^47^YMIVIGVII^55^	HLA-A*23:01, HLA-A*30:01, HLA-A*68:01	0.8039	NON-ALLERGEN
**E8L**			
^277^AIIAIVFVF^285^	A24, A26, B58, B62	0.7600	NON-ALLERGEN
^285^FILTAILFL^293^	A2, A26, A2, B8, B39, B62	0.5392	NON-ALLERGEN
^285^FILTAILFLM^294^	A1, B58, B62	0.4774	NON-ALLERGEN
^292^FLMSQRYSR^300^	A1, B58	0.9843	NON-ALLERGEN
^283^FVFILTAIL^291^	A1, A26, B62	0.4639	NON-ALLERGEN
^176^HSADAAWII^184^	A24, B62, A1, B27	0.8212	NON-ALLERGEN
^279^IAIVFVFIL^287^	A1, B58, B62	0.9039	NON-ALLERGEN
^290^ILFLMSQRY^298^	A2, B8	1.0469	NON-ALLERGEN
^215^ITENYRNPY^223^	A1, A26, B62	0.8011	NON-ALLERGEN
^291^LFLMSQRYSR^300^	A2, A26	0.8627	NON-ALLERGEN
^258^LREACFSYY^266^	A1, A26, B58, B62	1.5067	NON-ALLERGEN
^20^RLKTLDIHY^28^	HLA-A*01:01, HLA-A*02:06,HLA-A*02:01	1.9035	NON-ALLERGEN
^141^RSANMSAPF^150^	HLA-A*68:01, HLA-B*35:01, HLA-A*02:03, HLA-A*02:06, HLA-A*02:06	0.9595	NON-ALLERGEN
^177^SADAAWIIF^185^	HLA-A*23:01, HLA-B*58:01	0.8244	NON-ALLERGEN
^275^TFAIIAIVF^284^	HLA-A*68:01, HLA-B*53:01	1.1105	NON-ALLERGEN
^241^TTSPVRENY^250^	HLA-B*40:01, HLA-B*58:01	0.7917	NON-ALLERGEN
^122^VSDHKNVYF^131^	0HLA-A*23:01, HLA-A*30:02, HLA-B*44:03, HLA-A*11:01, HLA-A*33:01	1.0903	NON-ALLERGEN
^96^WNKKKYSSY^104^	HLA-A*02:01, HLA-A*02:03, HLA-A*03:01	0.7394	NON-ALLERGEN
^61^YVLSTIHIY^69^	HLA-A*68:02, HLA-A*30:02, HLA-A*68:01, HLA-A*02:06, HLA-A*23:01	0.5976	NON-ALLERGEN
^61^YVLSTIHIYW^70^	HLA-B*35:01, HLA-A*30:01, HLA-A*01:01	1.1449	NON-ALLERGEN
FVFILTAIL	B39, B62	0.4639	NON-ALLERGEN

**Table 5:** The overlapped HTL epitopes in Mpox virus glycoproteins.

HTL	Supertypes/HLA Alleles	Antigenicity score	Allergenicity
**A29L**			
^86^AETLRAAMISLAKKI^100^	HLA-DQA1*01:02/DQB1*06:02, HLA-DQA1*05:01/DQB1*03:01	0.5673	NON-ALLERGEN
^55^EKKITNITTKFEQIE^69^	HLA-DRB1*04:05, HLA-DQA1*03:01/DQB1*03:02	0.8686	NON-ALLERGEN
^56^KKITNITTKFEQIEK^70^	HLA-DPA1*02:01/DPB1*01:01, HLA-DPA1*02:01/DPB1*05:01, HLA-DQA1*04:01/DQB1*04:02, HLA-DPA1*01:03/DPB1*04:01	0.5631	NON-ALLERGEN
^54^LEKKITNITTKFEQI^68^	HLA-DRB1*04:05, HLA-DQA1*03:01/DQB1*03:02	0.7530	NON-ALLERGEN
^86^AETLRAAMISLAKKI^100^	HLA-DQA1*01:02/DQB1*06:02, HLA-DQA1*05:01/DQB1*03:01	0.5673	NON-ALLERGEN
^16^ATEFFSTKAAKNPET^30^	HLA-DPA1*02:01/DPB1*14:01, HLA-DRB1*09:01, HLA-DRB1*07:01, HLA-DPA1*02:01/DPB1*05:01, HLA-DQA1*04:01/DQB1*04:02, HLA-DRB3*02:02, HLA-DPA1*02:01/DPB1*01:01, HLA-DRB1*08:02, HLA-DPA1*03:01/DPB1*04:02, HLA-DRB5*01:01, HLA-DQA1*03:01/DQB1*03:02, HLA-DPA1*01:03/DPB1*04:01, HLA-DRB1*01:01, HLA-DRB1*04:01	0.3161	NON-ALLERGEN
^56^KKITNITTKFEQIEK^70^	HLA-DPA1*02:01/DPB1*01:01, HLA-DPA1*02:01/DPB1*05:01, HLA-DQA1*04:01/DQB1*04:02, HLA-DPA1*01:03/DPB1*04:01	0.5631	NON-ALLERGEN
^54^LEKKITNITTKFEQI^68^	HLA-DRB1*04:05, HLA-DQA1*03:01/DQB1*03:02	0.753	NON-ALLERGEN
**A35R**			
^68^AAITDSAVAVAAASS^82^	HLA-DQA1*01:02/DQB1*06:02, HLA-DQA1*05:01/DQB1*03:01	0.5513	NON-ALLERGEN
^65^ANEAAITDSAVAVAA^79^	HLA-DQA1*01:02/DQB1*06:02, HLA-DQA1*05:01/DQB1*02:01	0.54	NON-ALLERGEN
^155^DGNPITKTTSDYQDS^169^	HLA-DRB4*01:01, HLA-DRB1*04:01	0.6239	NON-ALLERGEN
^67^EAAITDSAVAVAAAS^81^	HLA-DQA1*01:02/DQB1*06:02, HLA-DQA1*05:01/DQB1*03:01	0.596	NON-ALLERGEN
^156^GNPITKTTSDYQDSD^170^	HLA-DRB4*01:01, HLA-DQA1*03:01/DQB1*03:02, HLA-DRB1*04:01	0.676	NON-ALLERGEN
^84^HRKVASSTTQYDHKE^98^	HLA-DQA1*03:01/DQB1*03:02, HLA-DQA1*04:01/DQB1*04:02, HLA-DRB1*04:05, HLA-DQA1*05:01/DQB1*02:01	0.8075	NON-ALLERGEN
^113^HSDYKSFEDAKANCA^127^	HLA-DQA1*01:01/DQB1*05:01, HLA-DRB1*04:05, HLA-DRB1*04:01	0.6016	NON-ALLERGEN
^70^ITDSAVAVAAASSTH^84^	HLA-DQA1*01:02/DQB1*06:02, HLA-DQA1*05:01/DQB1*03:01	0.5947	NON-ALLERGEN
^112^LHSDYKSFEDAKANC^126^	HLA-DQA1*01:01/DQB1*05:01, HLA-DRB1*04:05, HLA-DRB1*04:01	0.6088	NON-ALLERGEN
^66^NEAAITDSAVAVAAA^80^	HLA-DQA1*01:02/DQB1*06:02, HLA-DQA1*05:01/DQB1*03:01, HLA-DQA1*05:01/DQB1*02:01, HLA-DRB3*01:01	0.5949	NON-ALLERGEN
^85^RKVASSTTQYDHKES^99^	HLA-DQA1*03:01/DQB1*03:02, HLA-DQA1*04:01/DQB1*04:02, HLA-DRB1*04:05	0.7464	NON-ALLERGEN
^73^SAVAVAAASSTHRKV^87^	HLA-DRB5*01:01, HLA-DQA1*05:01/DQB1*03:01	0.7011	NON-ALLERGEN
^82^STHRKVASSTTQYDH^96^	HLA-DQA1*03:01/DQB1*03:02, HLA-DQA1*04:01/DQB1*04:02	0.5054	NON-ALLERGEN
^83^THRKVASSTTQYDHK^97^	HLA-DQA1*03:01/DQB1*03:02, HLA-DQA1*04:01/DQB1*04:02, HLA-DRB1*04:05	0.7592	NON-ALLERGEN
**L1R**			
^116^ESALATTAIDPVRYI^130^	HLA-DRB1*08:02, HLA-DQA1*05:01/DQB1*02:01, HLA-DRB1*13:02, HLA-DQA1*04:01/DQB1*04:02	0.5627	NON-ALLERGEN
^114^KKESALATTAIDPVR^128^	HLA-DPA1*01:03/DPB1*04:01, HLA-DRB3*01:01, HLA-DRB1*13:02	0.4051	NON-ALLERGEN
**M1R**			
^82^EQKAYVPAMFTAALN^96^	HLA-DRB1*13:02, HLA-DRB4*01:01, HLA-DRB4*01:01	0.4428	NON-ALLERGEN
^213^NDKIKLILANKENVH^227^	HLA-DRB1*13:02, HLA-DRB1*07:01, HLA-DPA1*02:01/DPB1*14:01, HLA-DPA1*01:03/DPB1*02:01	0.5600	NON-ALLERGEN
^222^NKENVHWTTYMDTFF^236^	HLA-DRB1*04:05, HLA-DRB1*04:05, HLA-DRB1*04:05, HLA-DRB1*03:01	0.4725	NON-ALLERGEN
^212^TNDKIKLILANKENV^226^	HLA-DQA1*05:01/DQB1*02:01, HLA-DPA1*02:01/DPB1*01:01	0.5444	NON-ALLERGEN
^121^VVDNKLKIQNVIIDE^135^	HLA-DPA1*01:03/DPB1*04:01, HLA-DPA1*03:01/DPB1*04:02	0.5877	NON-ALLERGEN
**E8L**			
^25^DIHYNESKPTTIQNT^39^	HLA-DRB1*09:01, HLA-DRB1*07:01	0.7124	NON-ALLERGEN
^209^EGKPHYITENYRNPY^223^	HLA-DPA1*01:03/DPB1*04:01, HLA-DPA1*02:01/DPB1*01:01	0.7163	NON-ALLERGEN
^47^FKGGYISGGFLPNEY^61^	HLA-DPA1*02:01/DPB1*01:01, HLA-DPA1*01:03/DPB1*04:01, HLA-DPA1*03:01/DPB1*04:02, HLA-DPA1*02:01/DPB1*05:01, HLA-DPA1*01:03/DPB1*02:01	0.6378	NON-ALLERGEN
^210^GKPHYITENYRNPYK^224^	HLA-DRB1*13:02, HLA-DPA1*01:03/DPB1*04:01, HLA-DPA1*03:01/DPB1*04:02, HLA-DPA1*02:01/DPB1*01:01, HLA-DPA1*01:03/DPB1*02:01,HLA-DPA1*02:01/DPB1*05:01	0.411	NON-ALLERGEN
^26^IHYNESKPTTIQNTG^40^	HLA-DRB1*09:01, HLA-DRB1*07:01	0.6046	NON-ALLERGEN
^48^KGGYISGGFLPNEYV^62^	HLA-DPA1*02:01/DPB1*01:01, HLA-DPA1*02:01/DPB1*05:01, HLA-DPA1*01:03/DPB1*04:01, HLA-DPA1*03:01/DPB1*04:02, HLA-DPA1*01:03/DPB1*02:01	0.6124	NON-ALLERGEN
^98^KKKYSSYEEAKKHDD^112^	HLA-DPA1*02:01/DPB1*05:01, HLA-DRB5*01:01	0.6188	NON-ALLERGEN
^211^KPHYITENYRNPYKL^225^	HLA-DRB1*13:02, HLA-DPA1*01:03/DPB1*04:01, HLA-DPA1*03:01/DPB1*04:02, HLA-DPA1*02:01/DPB1*01:01, HLA-DPA1*01:03/DPB1*02:01	0.5411	NON-ALLERGEN
^32^KPTTIQNTGKLVRIN^46^	HLA-DRB1*13:02, HLA-DRB1*03:01	0.5599	NON-ALLERGEN
^22^KTLDIHYNESKPTTI^36^	HLA-DRB3*02:02, HLA-DRB1*13:02	0.8991	NON-ALLERGEN
^24^LDIHYNESKPTTIQN^38^	HLA-DRB1*09:01, HLA-DRB1*07:01, HLA-DRB3*02:02	0.7166	NON-ALLERGEN
^21^LKTLDIHYNESKPTT^35^	HLA-DRB3*02:02, HLA-DRB1*13:02	0.8295	NON-ALLERGEN
^5^LSPINIETKKAISDA^19^	HLA-DRB1*13:02, HLA-DPA1*02:01/DPB1*05:01, HLA-DQA1*04:01/DQB1*04:02, HLA-DRB1*03:01	1.1086	NON-ALLERGEN
^46^NFKGGYISGGFLPNE^60^	HLA-DPA1*02:01/DPB1*01:01, HLA-DPA1*01:03/DPB1*04:01, HLA-DPA1*03:01/DPB1*04:02, HLA-DPA1*01:03/DPB1*02:01, HLA-DPA1*02:01/DPB1*05:01	0.7965	NON-ALLERGEN
^97^NKKKYSSYEEAKKHD^111^	HLA-DPA1*02:01/DPB1*05:01, HLA-DRB5*01:01, HLA-DPA1*02:01/DPB1*01:01	0.6247	ALLERGEN
^53^SGGFLPNEYVLSTIH^67^	HLA-DPA1*01:03/DPB1*04:01, HLA-DPA1*01:03/DPB1*02:01	0.4959	NON-ALLERGEN
^6^SPINIETKKAISDAR^20^	HLA-DQA1*04:01/DQB1*04:02, HLA-DRB1*13:02, HLA-DPA1*02:01/DPB1*05:01, HLA-DRB1*08:02	1.032	NON-ALLERGEN
^23^TLDIHYNESKPTTIQ^37^	HLA-DRB1*09:01, HLA-DRB1*13:02, HLA-DRB3*02:02, HLA-DRB1*07:01	0.7176	NON-ALLERGEN
^96^WNKKKYSSYEEAKKH^110^	HLA-DPA1*02:01/DPB1*05:01, HLA-DPA1*02:01/DPB1*01:01	0.4512	NON-ALLERGEN

**Table 6:** Antigenicity score and allergenicity score of selected continuous B-cell epitopes present on the surface of target proteins.

Proteins	Epitopes	Antigenicity score	Antigenicity	Allergenicity
A30L	^30^YGNIKEFNATHAAFEYSKSIGGTPAL DRRVQDVNDTISDVKQK^72^	0.8069	ANTIGEN	NON-ALLERGEN
A35R	^67^EAAITDSAVAVAAASSTHRKVA SSTTQYDHKESCN^101^	0.6612	ANTIGEN	NON-ALLERGEN
A35R	^113^HSDYKSFE^120^	0.5871	ANTIGEN	NON-ALLERGEN
A35R	^131^STLPNKSDVL^140^	0.7299	ANTIGEN	NON-ALLERGEN
A35R	^147^YVEDTWGSDGNPITKTTSDYQD SDVSQEVRKY^178^	0.4725	ANTIGEN	NON-ALLERGEN
L1R	^114^KKESALATTAID^125^	0.9842	ANTIGEN	NON-ALLERGEN
M1R	^23^EQEANASAQT^32^	0.5889	ANTIGEN	NON-ALLERGEN
M1R	^41^FYIRQNHG^48^	0.4708	ANTIGEN	NON-ALLERGEN
M1R	^79^LTPEQKAY^86^	1.4971	ANTIGEN	NON-ALLERGEN
M1R	^169^KATTQIAPRQVAGT^182^	0.6607	ANTIGEN	NON-ALLERGEN
E8L	^26^IHYNESKP^33^	0.4582	ANTIGEN	NON-ALLERGEN
E8L	^98^KKKYSSYEEAKKH^110^	0.4493	ANTIGEN	NON-ALLERGEN
E8L	^203^LSSSNHEGKPHYITENYRN PYKLND^227^	0.5836	ANTIGEN	NON-ALLERGEN

**Table 7:** Discontinuous B-cell epitopes present on the surface of target proteins.

Protein	Residues	Number of residues	Score
A29L	A:F6, A:P7, A:G8, A:D9, A:D10, A:D11, A:L12, A:A13	8	0.901
	A:I100, A:Q103, A:T104, A:G105, A:R106	5	0.84
A30L	A:M1, A:N2, A:S3, A:L4, A:S5, A:I6, A:F7, A:F8, A:I9, A:V10, A:V11, A:A12, A:T13	13	0.932
	A:A41, A:A42, A:F43, A:E44, A:Y45, A:S46, A:K47, A:S48, A:I49, A:T52	10	0.853
A35R	A:M2, A:T3, A:P4, A:E5, A:N6, A:D7, A:E8, A:E9, A:Q10, A:T11, A:S12, A:V13, A:F14, A:S15, A:A16, A:T17	16	0.896
	A:G156, A:N157, A:T160, A:K161, A:T162, A:T163, A:S164, A:D165, A:Y166, A:Q167, A:D168, A:S169, A:D170, A:V171, A:S172, A:Q173, A:E174	17	0.858
L1R	A:L55, A:E56, A:A57, A:V58, A:G59, A:H60, A:C61, A:Y62, A:E63	9	0.919
	A:D135, A:I136, A:A137, A:N140, A:D143, A:I144, A:S147, A:N148, A:V150, A:E151, A:K152	11	0.885
M1R	A:M1, A:G2, A:A3, A:A4, A:A5, A:S6, A:I7, A:Q8, A:Y76, A:S77, A:G78, A:L79, A:T80, A:P81, A:E82, A:Q83, A:K84	17	0.906
	A:L218, A:I219, A:L220, A:A221, A:N222, A:K223, A:E224, A:N225, A:V226, A:H227, A:W228, A:T229, A:T230, A:Y231, A:M232, A:D233, A:T234, A:F236, A:R237	19	0.903
	A:T210, A:N213, A:D214, A:K215, A:I216, A:K217	6	0.857
	A:G138, A:A139, A:P140, A:G141, A:S142	5	0.819
E8L	A:K268, A:Y269, A:E271, A:G272, A:N273, A:K274, A:T275, A:F276, A:A277, A:I278, A:I279, A:A280, A:I281, A:V282, A:F283, A:V284, A:F285, A:I286, A:L287, A:T288, A:A289, A:I290, A:L291, A:F292, A:L293, A:M294, A:S295, A:Q296, A:R297, A:Y298, A:S299, A:R300, A:K302, A:Q303, A:N304	35	0.92
	A:R142, A:S205, A:S206, A:N207, A:H208, A:E209, A:G210, A:K211, A:P212, A:H213, A:Y214	11	0.846
	A:L225, A:N226, A:D227, A:D228, A:T229, A:Q230, A:V231	7	0.832
	A:P58, A:N59, A:E60, A:K98, A:K99, A:K100, A:Y101, A:S102, A:S103, A:E106, A:K109, A:H110, A:D112	13	0.802

**Fig. 1:**
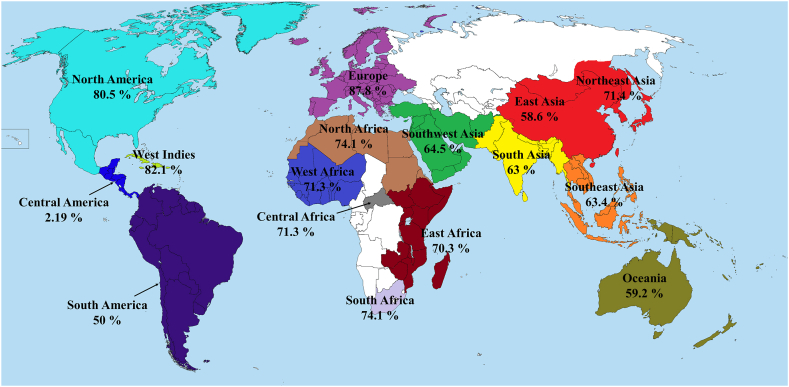
Population Coverage. The plot of HLA population coverage of designed vaccine across different regions.

The Mpox virus hinders the normal functioning of natural killer (NK) cells, leading to a decrease in the secretion of important immune response molecules, such as IFN-γ and TNF-α [12]. This impairment occurs through the virus's ability to inhibit the expression of chemokines (CCR5, CXCR3, and CCR6), which play crucial roles in the immune system [13]. During Mpox infection, the activation of IFN-γ-producing Th, Tc, and NK cells are essential for triggering protective immunity through the stimulation of cell-mediated immune responses. To achieve this, we have utilized the IFN epitope server to predict IFN-γ epitopes for each specified target glycoprotein. After evaluating their immunogenicity, we selected a total of 5 IFN-γ epitopes to incorporate into the vaccine design (Table 8). In the process of constructing the final vaccine, the Tc epitopes underwent further screening using population coverage analysis and conservation analysis. Four Tc epitopes (^87^SIFGFQAEV^95^, ^50^TMSAFLIVR^58^, ^20^RLKTLDIHY^28^, and ^96^WNKKKYSSY^104^) demonstrated a global coverage exceeding 50 % and 100 % conservancy among all glycoproteins, which were subsequently chosen for the final vaccine design (Table 9). These selected Tc epitopes were found to overlap with three Th epitopes (^96^WNKKKYSSYEEAKKH^110^, ^22^KTLDIHYNESKPTTI^36^, and ^21^LKTLDIHYNESKPTT^35^), which were selected in the final vaccine design and construct (Table 10). The selected Tc and Th specific epitopes showed substantial human leukocyte antigen (HLA) coverage in the different regions of the world such as North America, North Africa, Europe, Africa, and Asia, suggested that the immunogenicity of those epitopes have great prospect for efficiently providing protection against Mpox infection on a global scale (Fig. 1).

**Table 8:** List of highest antigenic IFN-γ epitopes predicted by IFN epitope server.

Protein	Epitope	Method	Result	Score	Antigenicity Score	Antigenicity	Allergenicity
A29L	^46^TLKQRLTNLEKKITN^60^	SVM	POSITIVE	0.8679	0.7991	ANTIGEN	NON-ALLERGEN
A30L	^61^DVNDTISDVKQKWRC^75^	MERCI	POSITIVE	5.0000	1.5276	ANTIGEN	NON-ALLERGEN
A35R	^31^RVIGLCIRISMVISL^44^	SVM	POSITIVE	1.0136	1.5629	ANTIGEN	NON-ALLERGEN
L1R	^121^TTAIDPVRYIDPRRD^135^	SVM	POSITIVE	0.1259	0.6472	ANTIGEN	NON-ALLERGEN
M1R	^31^QTKCDIEIGNFYIRQ^45^	SVM	POSITIVE	0.2754	1.2368	ANTIGEN	NON-ALLERGEN

**Table 9:** Details of CTL epitopes with their population coverage efficiency.

S. No	Protein	CTL	Population coverage
1	A30L	^87^SIFGFQAEV^95^	61.93 %
2	A35R	^50^TMSAFLIVR^58^	51.06 %
3	E8L	^20^RLKTLDIHY^28^	53.77 %
4	E8L	^96^WNKKKYSSY^104^	52.70 %

**Table 10:** The CTL epitopes overlapped with HTL epitopes.

S. No	Protein	HTL	CTL
1	E8L	^96^WNKKKYSSYEEAKKH^110^	WNKKKYSSY
2	E8L	^22^KTLDIHYNESKPTTI^36^	RLKTLDIHY
3	E8L	^21^LKTLDIHYNESKPTT^35^	RLKTLDIHY

### 2.3 Construction of vaccine

The final design of the multi-epitope hybrid includes four Tc-cells activating epitopes, three Th-cells activating epitopes, thirteen B-cells activating epitopes, and five IFN-γ secreting epitopes. To enhance immunogenicity, we added the PADRE peptide (AKFVAAWTLKAAA) as an adjuvant at the N-terminus of the vaccine sequence. The EAAAK peptide served as a linker to connect the adjuvant with the Th-cells epitopes. For the vaccine construct, each Th-cells epitope, B-cells epitope, and IFN-γ epitope were linked together using the GPGPG peptide, while Tc-cells epitopes were connected with the AAY peptide. The resulting vaccine sequence comprises 534 amino acids and weighs 55 kDa (Table 11, Table 12). Importantly, this multi-epitope hybrid vaccine exhibits strong immunogenicity and lacks any allergic characteristics.

**Table 11:** The sequence of Mpox vaccine, their antigenicity score, and allergenicity.

Sequence	Size (aa)	Antigenicity (>0.4)	Allergenicity
AKFVAAWTLKAAAEAAAKYGNIKEFNATHAAFEYSKSIGGTPALGPGPGDRRVQDVNDTISDVKQKGPGPGEAAITDSAVAVAAASSTHRKVAGPGPGSSTTQYDHKESCNGPGPGHSDYKSFEGPGPGSTLPNKSDVLGPGPGYVEDTWGSDGNPITKTTSDYQDGPGPGSDVSQEVRKYGPGPGKKESALATTAIDGPGPGEQEANASAQTGPGPGFYIRQNHGGPGPGLTPEQKAYGPGPGKATTQIAPRQVAGTGPGPGIHYNESKPGPGPGKKKYSSYEEAKKHGPGPGLSSSNHEGKPHYITENYRNGPGPGPYKLNDGPGPGSIFGFQAEVAAYTMSAFLIVRAAYRLKTLDIHYAAYWNKKKYSSYGPGPGWNKKKYSSYEEAKKHGPGPGKTLDIHYNESKPTTIGPGPGLKTLDIHYNESKPTTGPGPGTLKQRLTNLEKKITNGPGPGDVNDTISDVKQKWRCGPGPGRVIGLCIRISMVISLGPGPGTTAIDPVRYIDPRRDGPGPGQTKCDIEIGNFYIRQ	534	0.5775 Probable Antigen	Probable Non-Allergen

**Table 12:** Physiochemical properties of Mpox vaccine.

Physiochemical properties	
Molecular weight	55630.76
Theoretical pI	9.14
Estimated half-life	
1. mammalian reticulocytes, *in vitro*	4.4 h
2. yeast, *in vivo*	>20 h
3. *Escherichia coli*, *in vivo*	>10 h
Instability Index	27.36 (protein as stable)
Aliphatic index	51.78
Grand average of hydropathicity (GRAVY)	−0.805
Solubility in *Escherichia coli.* (>0.5)	0.506

### 2.4 Characterization of vaccine

The designed vaccine is found to be immunogenic. Initially, we have investigated the physio-chemical properties of the vaccine (Table 12). Theoretical isoelectric point (pI) analysis revealed that the vaccine has a pI of 9.14, indicating its alkaline nature. The calculated half-life of the vaccine was observed to be 4.4 h in mammalian reticulocytes, exceeding 20 h in yeast, and surpassing 10 h in *E. coli*. The vaccine exhibited an instability index of 27.36, suggesting high stability of the protein. Further details regarding the aliphatic index, GRAVY, and solubility of the vaccine can be found in Table 12. The GRAVY score of the designed vaccine is −0.805. This negative GRAVY score indicates that the vaccine protein is hydrophilic, suggesting good solubility in aqueous solutions. This information implies that the vaccine is likely to be stable and effectively interact with the immune system, enhancing its overall efficacy.

### 2.5 3D configuration of vaccine

**Fig. 2:**
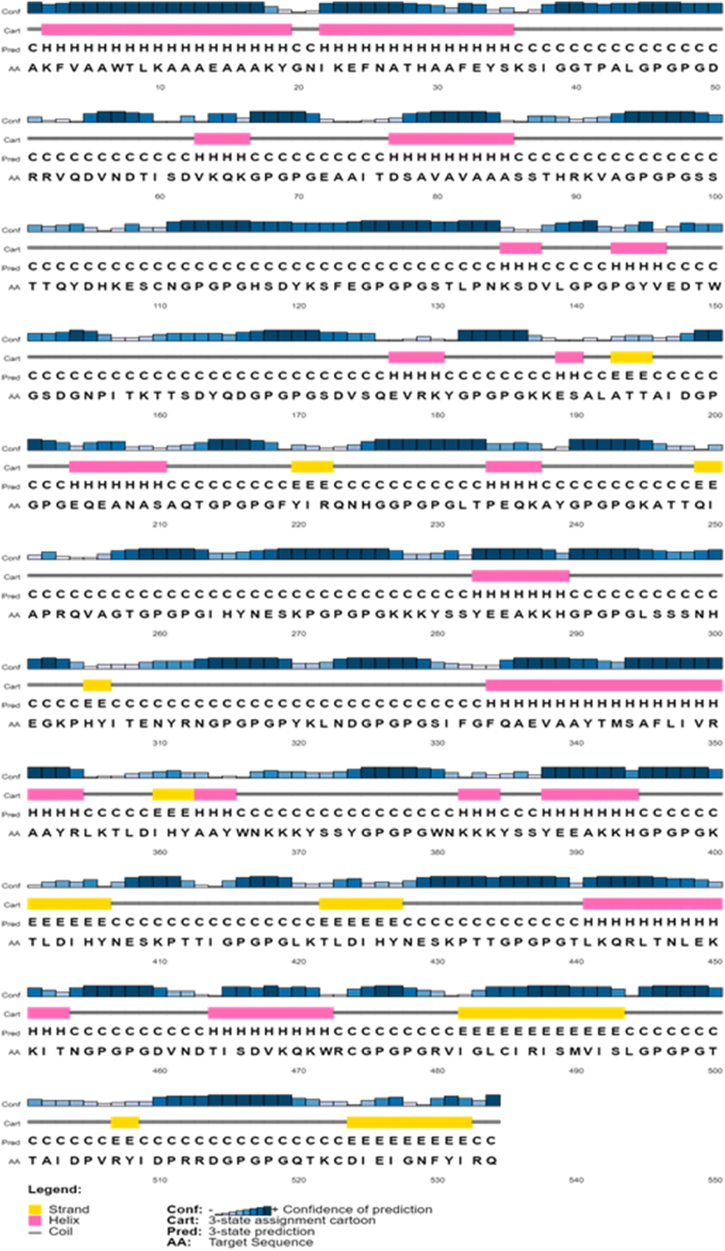
Secondary structure components of Mpox vaccine. The per residue β-strand, helix and coil content present in the vaccine is shown in yellow, pink and black color, respectively.

**Fig. 3:**
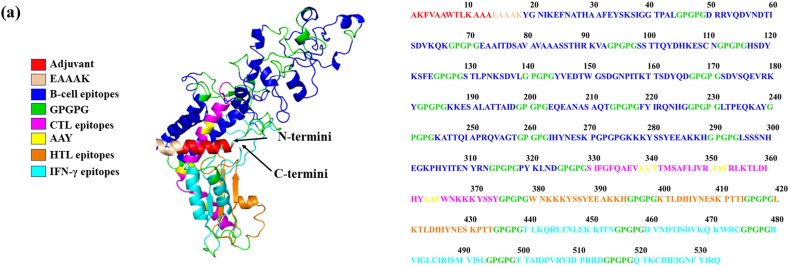
3D structure representing the final configuration of the vaccine. In the visualization, distinct colors highlight the adjuvant, various epitopes, and linkers. The amino acid sequence of the vaccine is comprised of 534 residues, underscoring the intricacy of its molecular composition.

The secondary structural elements of the designed vaccine were analysed using the SOPMA online tool. We illustrated the different secondary structure elements, including β-strands, helices, coils, and others, each represented by distinct colors (Fig. 2). Afterward, the vaccine sequence was submitted to three homology servers: I-tasser, Robetta, and IntFOLD, in order to generate 3D structures [[14], [15], [16]]. To ensure accuracy, the obtained 3D structures underwent refinement using the Galaxy refine server, aiming to minimize any distortions in the modelled structures [17]. The validation of each model of vaccine was done by Ramachandran plot using PROCHECK server [18]. Notably, the structure obtained from the Robetta server, post-refinement, exhibited only 1.13 % of Rama outliers and 98.3 % of Rama favoured regions, surpassing the other homology servers' performance (Table 13). The final 3D structure of vaccine is shown in Fig. 3.

**Table 13:** Ramchandran plot of Mpox vaccine.

I-tasser			Robetta			IntFOLD		
Rama allowed >99.8 %	Rama outliers <0.05 %	Rama favoured >98 %	Rama allowed >99.8 %	Rama outliers <0.05 %	Rama favoured >98 %	Rama allowed >99.8 %	Rama outliers <0.05 %	Rama favoured >98 %
95.9	4.14	81.02	**98.9**	**1.13**	**95.86**	98.3	1.69	93.98

### 2.6 Molecular dynamics (MD) studies of vaccine-immune receptor interaction

**Fig. 4:**
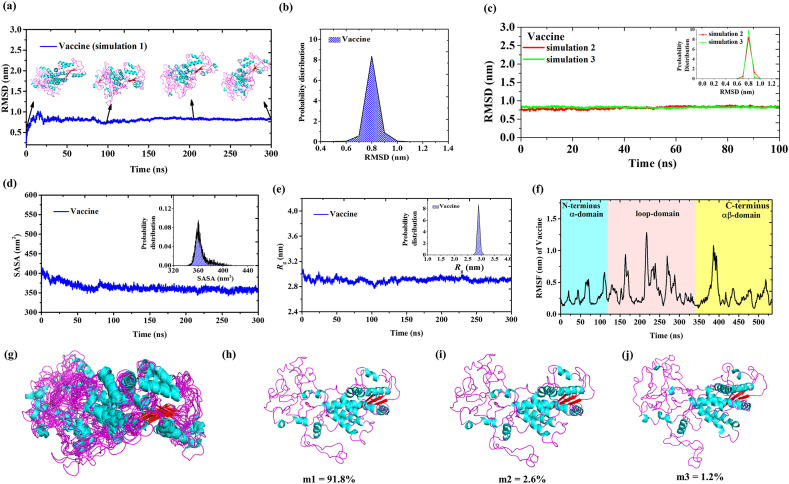
MD simulation of Mpox vaccine. (a) Time-dependent graph displaying root mean square deviation (RMSD) of the vaccine. Four snapshots at distinct time points: 0 ns, 100 ns, 200 ns, and 300 ns are indicated in cartoon representation. (b) Probability distribution of RMSD of Vaccine (c) RMSD of simulation 2 & 3 of vaccine (d) Time dependent SASA of vaccine with its probability distribution graph in the inset (e) Time dependent graph of *R*_g_ with its probability distribution graph in the inset (f) RMSF (g) Superimposed structures of vaccine at various time points (0, 50, 100, 150, 200, 250 and 300 ns) and (h–j) the most-population microstates m1, m2 and m3 of vaccine simulation. The percentage indicates the population of that microstates w.r.t to total number of conformations.

MD simulations have proven useful in evaluating the stability of the protein's structure and detecting potential structural modifications, including protein-protein interactions and conformational dynamics. These simulations will help to provide insights into the flexibility of the vaccine and its interactions with receptors. In this study, we conducted a 300 ns MD simulation of the vaccine using GROMACS and employed various built-in tools to assess its properties. The evaluation involved analysing the root mean square deviation (RMSD), solvent accessible surface area (SASA), the radius of gyration (*R*_g_), and root mean square fluctuation (RMSF) of the vaccine (Fig. 4b–d). Importantly, the vaccine's RMSD trajectory exhibited remarkable stability throughout the entire simulation phase, indicating minimal changes in atom positions over the duration of the study. (Fig. 4a). The probability distribution graph of RMSD gives an average value of 0.80 ± 0.01 nm, which indicated the high stability of vaccine structure (Fig. 4b). To verify the convergence of the simulation, we conducted two additional simulations, each spanning 100ns, as illustrated in Fig. 4c. These simulations, labelled as 2 and 3, mirror the RMSD of simulation 1, affirming the reproducibility of the MD results.

Further, a saturated curve was observed in the time-dependent SASA of vaccine with average value of 364.08 ± 4.75 nm^2^ (Fig. 4d). The *R*_g_ of the vaccine during simulation exhibited an average value of 2.90 ± 0.003 nm (Fig. 4e). The single sharp peak in the probability distribution graph suggested compactness and stability of vaccine (inset). The structure of vaccine exhibited three domains: α-domain, loop domain and αβ-domain Furthermore, the RMSF analysis revealed that regions between 100 and 300 residues of loop domain and 350–400 residues of the αβ-domain of vaccine exhibited greater flexibility compared to the N-terminus and C-terminus regions (Fig. 4f). Later, the vaccine conformations at various time points (0, 50, 100, 150, 200, 250, and 300 ns) were superimposed, as depicted in Fig. 4g. The different conformations of vaccine didn't show much change in structure during simulation. The clustering technique has been employed for conformational sampling of vaccine. The three most–populated microstates of vaccine simulation were shown in Fig. 4h-j. The three microstates m1, m2 and m3 contributes 95.6 % of total population of the simulation, which indicates the designed vaccine was structurally stable protein.

### 2.7 Vaccine-receptor binding analysis and their dynamic behaviour

**Fig. 5:**
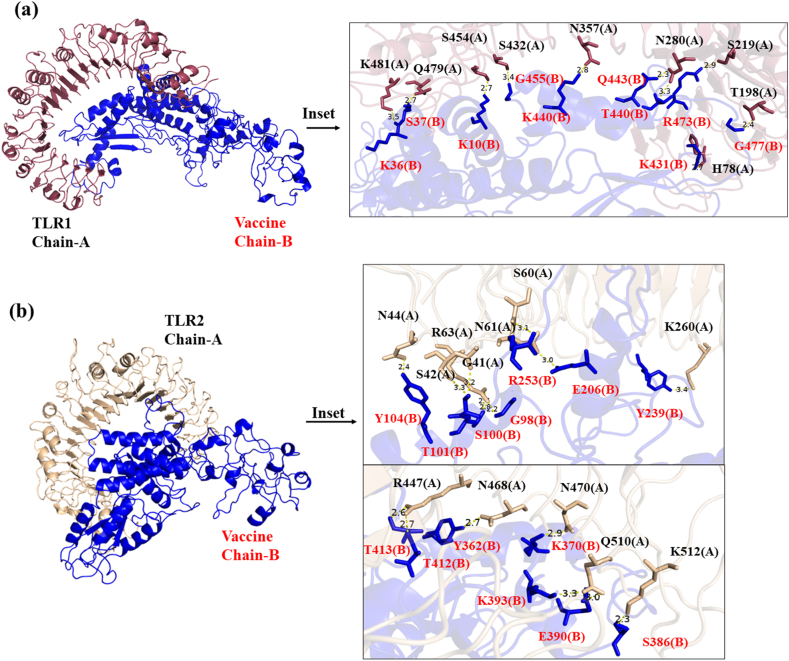
The molecular docking of vaccine with (a) TLR1 receptor (b) TLR2 receptor of human. These docking simulations provide insights into the molecular interactions between the vaccine and human TLR1 or TLR2, highlighting their potential roles in initiating immune responses.

**Fig. 6:**
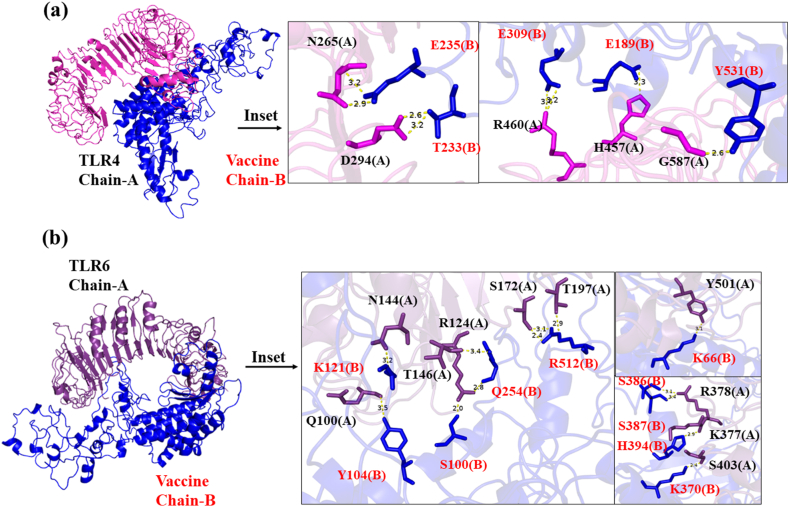
The molecular docking of multi-epitope vaccine with (a) TLR4 receptor (b) TLR6 receptor of human. Molecular docking of the vaccine with the TLR4 and TLR6 demonstrates specific and favorable noncovalent binding interactions, elucidating the potential activation of immune responses.

**Fig. 7:**
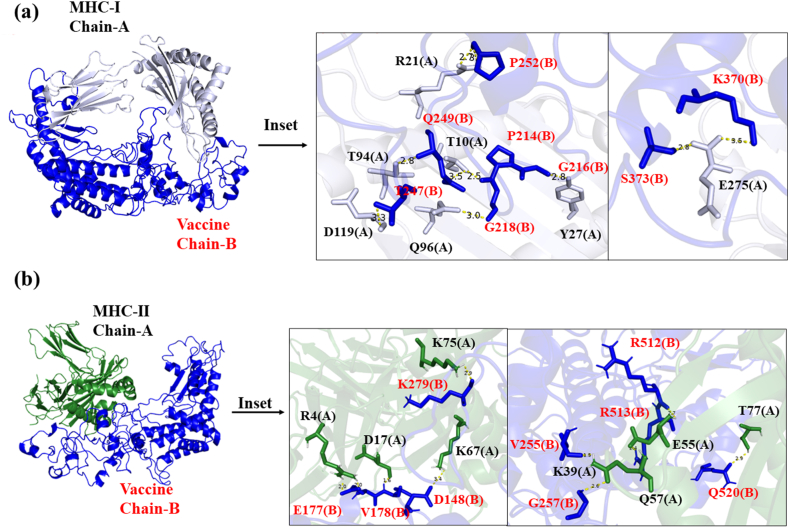
The molecular docking showing binding of Mpox vaccine with (a) MHC-I (b) MHC-II of human. Molecular docking of the vaccine with MHC I and II reveals precise and favorable binding interactions, offering insights into potential immune response activation mechanisms. *The two chains of MHC-II are denoted by chain A = (a); chain B= (b)*.

TLRs are the integral components of the innate immune system serving a crucial function in detecting pathogens and triggering adaptive immune responses [19]. The importance of TLRs in vaccine development stems from their capacity to stimulate innate immunity and improve antigen presentation, thus contributing to the creation of highly effective vaccines. We have checked the binding interactions of the designed multi-epitopes vaccine with various pathogen recognition receptors like TLRs (TLR1, TLR2, TLR4, TLR6), MHC-I and MHC-II receptor by using online webserver ClusPro. Out of them, vaccine showed maximum binding efficiency with TLR1 and TLR6 (Table 14). The seven amino acids residues (S454, N280, S219, Q479, H78, T198, K456) of TLR1 are involved in forming hydrogen binding interaction with seven residues (K10, T440, R473, S37, K431, G477, E14) of vaccine and 26 residues of TLR1 form hydrophobic contacts with 25 residues of Vaccine, which leads to the most favorable binding energy (ΔG = −20 kcal mol^−1^) and lowest K_d_ = 6.1e-16 M value (Fig. 5a and Table 14, S2). In case of TLR6-Vaccine complex, the ten residues (Q100, N144, T146, R124, S172, T197, Y501, R378, K377 and S403) of TLR6 involved in hydrogen bonding with ten residues (K121, Y104, S100, Q254, R512, K66, S386, S387, H394 and K370) of vaccine, contributes to favorable binding energy. The molecular docking results of vaccine with the other receptors are clearly described in Fig. 5, Fig. 6, Fig. 7 and Table 14. The best binding pose of vaccine with all receptors are chosen for molecular dynamics studies. The residues of TLRs & MHC-I/II which are involved in hydrogen bonding and hydrophobic contacts with vaccine are clearly mentioned in Table S3.

**Table 14:** The binding energy and K_d_ of Mpox vaccine with TLR receptors and MHC-I, MHC-II.

TLR receptors	ΔG (kcal mol^−1^)	K_d_ (M) at 25 °C
TLR1	−20.7	6.1e-16
TLR2	−14.8	1.3e-11
TLR4	−17.0	3.6e-13
TLR6	−19.8	3e-15
MHC-I	−9.1	2.1e-07
MHC-II	−12.9	3.7e-10

**Fig. 8:**
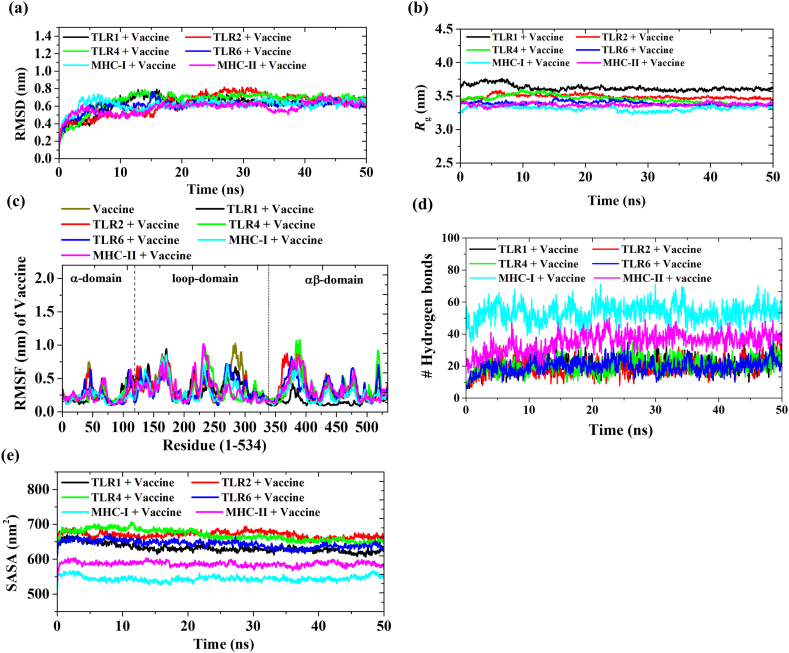
MD simulation of Mpox vaccine with TLR1, TLR2, TLR4, TLR6, MHC-I and MHC-II. (a) RMSD (b) Rg (c) RMSF (d) Number of hydrogen bond between Mpox vaccine and various receptors (TLR1, TLR2, TLR4, TLR6, MHC-I and MHC-II) (e) SASA plot showing the changes in solvent-accessible surface area of the vaccine and immune receptors.

A total of six systems were subjected for 50 ns MD simulation; (i) TLR1 + Vaccine, (ii) TLR2 + Vaccine, (iii) TLR4 + Vaccine, (iv) TLR6 + Vaccine, (v) MHC-I + Vaccine and (vi) MHC-II + Vaccine. The RMSD of the vaccine is fluctuated in the presence of all receptors (Fig. 8 and Table 15). Notably, a significant change in the RMSD value of the vaccine occurred when the TLR4 receptor was present (increasing from 0.751 nm to 0.848 nm), thereby emphasizing the strong interaction between TLR4 and the vaccine (Fig. 8a–S1 and Table 15). The structural stability of the vaccine persists in the presence of other receptors, resulting in a diminished RMSD value. The R_g_ of the vaccine was calculated in the presence of receptors. The R_g_ value of the vaccine increased by approximately >1 nm in the presence of TLR receptors, while an increase of >3 nm was observed for MHC-I and MHC-II receptors (Fig. 8b–S1 and Table 15). A higher R_g_ value of vaccine suggested that the vaccine get more flexible in the presence of receptor. The RMSF value of vaccine in presence of different receptors was estimated (Fig. 8c). In presence of TLR1, the RMSF value in N-terminus region (0–56 residues) and 350–534 region of vaccine is significantly fluctuated as compared to vaccine alone, indicated the binding region of TLR1 with vaccine. RMSF of vaccine has fluctuated majorly within 1–300 residues region in the presence of TLR4 and TLR6. MHC-I and MHC-II affect the N-terminus and mid-region (200–250 residues) of the vaccine structure (Fig. 8c). Hydrogen bonds play a crucial role in molecular recognition and binding interactions. An increased count of hydrogen bonds signifies a more robust binding affinity between the vaccine and the receptor. TLR4 showed a larger number of hydrogen bonds with the vaccine compared to other TLR receptors. Among the MHC-I and MHC-II, vaccine showed 1.4 times more hydrogen bonding interaction with MHC-I compared to MHC-I during simulation (Fig. 8d–S1 and Table 15).

**Table 15:** The average RMSD, *R*_g_ and number of hydrogen bonds between Mpox vaccine and receptor.

System	Average RMSD	Average *R*_g_	Average SASA	Average number of hydrogen bonds between vaccine and receptors
Vaccine+^TLR1^	0.623 ± 0.06	3.618 ± 0.02	632.67 ± 4.98	20.4
Vaccine + TLR2	0.627 ± 0.06	3.489 ± 0.01	669.91 ± 5.76	19.3
Vaccine + TLR4	0.664 ± 0.08	3.454 ± 0.02	669.23 ± 5.76	20.5
Vaccine + TLR6	0.605 ± 0.07	3.387 ± 0.01	644.03 ± 2.36	19.6
Vaccine + MHC-I	0.619 ± 0.03	3.313 ± 0.003	544.73 ± 2.93	53.5
Vaccine + MHC-II	0.582 ± 0.03	3.369 ± 0.003	586.85 ± 1.13	35.0

**Fig. 9:**
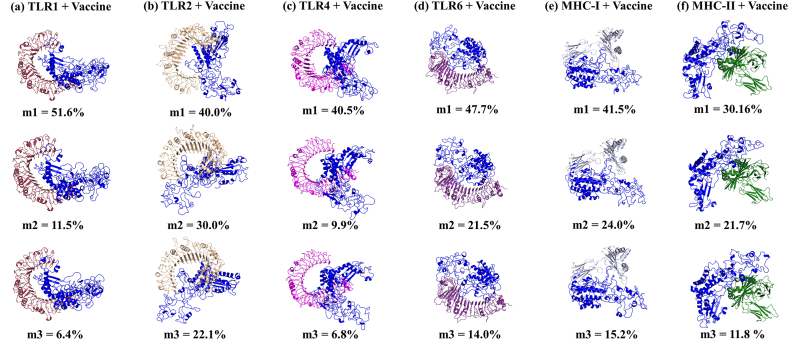
Clustering. The three most populated conformations (m1, m2 and m3) of six MD simulation systems (a) TLR1 + Vaccine; (b) TLR2 + Vaccine; (c) TLR4 + Vaccine; (d) TLR6 + Vaccine; (e) MHC-I + Vaccine; (f) MHC-II + Vaccine. The percentage indicates the population of the microstates (m1, m2 and m3) w.r.t to total number of conformations.

The clustering technique has been employed for conformational sampling of vaccine-receptor simulation. The three most populated microstates m1, m2 and m3 of all six simulations (TLR1 + Vaccine; TLR2 + Vaccine; TLR4 + Vaccine; TLR6 + Vaccine; MHC-I + Vaccine; MHC-II + Vaccine) contributes 69.1 %, 92.1 %, 57.3 %, 83.1 %, 80.6 % and 62.5 % of the population of whole trajectory (Fig. 9). The interaction of vaccine with human TLRs and MHC's receptors during MD simulation are shown in Movie 1-6. In Vaccine + TLR1 simulation, B-cell, IFN-γ and Th-cells epitopes of vaccine participated in bonding with TLR1 receptor through hydrogen bonding and hydrophobic interaction (Movie 1, Table 16). Whereas, in presence of TLR2, dominantly B-cell epitopes and few Th-cells epitopes showed interaction with TLR2 receptor (Movie 2, Table 16). Similarly, presence of TLR4, MHC-II receptor, B-cell epitopes and IFN-γ epitopes of vaccine responsible for binding with these receptors (Movie 3, 6 & Table 16). In case of TLR6 and MHC-I, Th-cells and B-cell epitopes of vaccine were participating in the molecular interactions with receptors (Movie 4, 5 & Table 16). The detail of the residues of vaccine involved in hydrogen bonding and hydrophobic contacts with receptors are given in Table 16. In SASA analysis, the complex formation of vaccine with TLR receptors showed maximum interaction with solvent followed by MHC receptors in the order: TLR2>TLR4>TLR6>TLR1>MHC-II > MHC-I Fig. 8e–S1, Table 16). The large shift in the average SASA value with a gradual descending trend suggests conformational changes in the accessibility of specific regions, potentially impacting the strength and specificity of the binding between vaccines and receptors. Further, the convergence of simulations was tested by performing one additional simulations with different initial velocity for all six systems. The RMSD curves for simulation 2 is analogous to that for simulation 1 in all systems [Fig. S2 (a-f)]. This indicates the convergence of all trajectories and reproducibility of MD results. Overall, the interaction studies between the vaccine and receptors highlighted that the vaccine possesses a strong binding affinity among multiple TLRs and MHC molecules of human, which will trigger the immune response and leads to the activation of innate and adaptive immune systems.

**Table 16:** The binding interaction between the vaccine and receptors in most populated microstate m1 during simulation.

System	Residue involved in hydrogen bonding		Residues involved in hydrophobic interactions	
	Vaccine (Chain B)	Receptor (Chain A)	Vaccine (Chain B)	Receptor (Chain A)
Vaccine+^TLR1^	H289	N519	T434, W7, H305, G455, P456, H300, P458, T195, P476, *P*432, P438, S387, Y388, F334, A391, K382, I38, W380, P398, Y385, P396, K383	S101, T501, S540, M381, S432, G535, K530, P537, R539, H453, S534, F123, H78, N252, N34, K33, G533, G35, I37, P503, Y56, H38
	E309	K536		
	G457	Q407		
	K450	N357		
	N299	S528		
	Q443	N280		
	G475	H102		
	K431	H102		
	G399	Q54		
	T401	Q54		
	K10	S454		
	N381	R80		
Vaccine + TLR2	Q65	Y323	Y388, P411, K410, G67, P68, G69, P217, V178, F123, P241, Q176, I250, P252, S100, K66	H449, F322, S427, S424, H426, V82, F349, Y376, V373, F128, I153, S45, Y66, T65, N44, Y109, P47, S40, N61, L371
	Q65	P320		
	S409	R447		
	P416	R447		
	T248	D106		
	T247	R155		
	D62	K347		
	D105	R63		
	E124	R63		
	Y104	S42		
	Y239	Y111		
	P70	H398		
	S175	S48		
	A251	D58		
	Q254	S39		
	Q254	G41		
	R253	G41		
	R253	S60		
	R253	N62		
	R253	G38		
Vaccine + TLR4	N299	K533	V461, P42, I38, G39, F334, H305, G329, L192, Q335, W7, P46, G47, I331, K23, I22, A16, A17,G20, V63, P68, K66, K64, H300	P589, G587, Q430, S529, Q505, H456, G480, F408, G579, R382, A528, W550, S504, N433, M41, Q39, V33, V32, T37, F63, N64, E89, P65, R87, P555, Q531
	E284	K533		
	N462	K582		
	P458	K582		
	Q534	S580		
	Q534	T553		
	K287	E509		
	S297	E509		
	S297	N486		
	A191	Q507		
	T195	Q507		
	S330	H458		
	S190	H458		
	Q236	S360		
	Q236	N339		
	Q236	K362		
	E235	K362		
	S37	H552		
	S37	N526		
	T41	H431		
Vaccine + TLR6	E389	R378	P217, F219, G214, P215, Y120, Y362, Y365, R512, R253, P252, Y104, A246, Q249, G67, T101, H361, E390, K121, S122, F123,	C173, V198, Q257, H222, T255, T146, A474, K453, Q476, S498, Q473, T149, S172, K147,S122,A56,S34,M35,Y104, R82, V537, R124, Q500, S403, N144, H168, L164, P165
	G216	Q224		
	Q254	H170		
	Q254	L148		
	T248	E33		
	T247	E33		
	E72	R531		
Vaccine + MHC-I	A251	R21	R252, P217, G218, Y220, G415, G71, G214,Q254, Y104, T248, I250	F8, Y27, V25, I23, H191, V194, R234, A41, A40, G18, G120, D119, S92, T94
	P252	R21		
	I414	H192		
	T247	K121		
	T413	A193		
	R512	D39		
	D105	R17		
	Q249	Q96		
Vaccine + MHC-II	S172	R4	P170, G171, Q176, C523, T521, P516, G497, G294, L295, Y283, S282, E301, G259, P260, D324, S282, G294, R513, Y280, I264, G257, A256, V255, G263, G261, P262	P5, T3, V75, A73, H81, E59, P56, L67, V65, A61, I63, G58, I72, F54, S53, Y79, Q57, A68,A64
	K522	R72		
	K522	D76		
	Q520	T77		
	Q520	Q70		
	E284	R55		
	S297	Q64		
	S297	Y60		
	G302	D66		
	Y306	N62		
	S281	R76		
	R512	E55		
	R512	E40		
	K277	K75		
	G276	K75		
	K279	E71		
	K180	Q18		
	D148	Q18		

### 2.8 PCA analysis

**Fig. 10:**
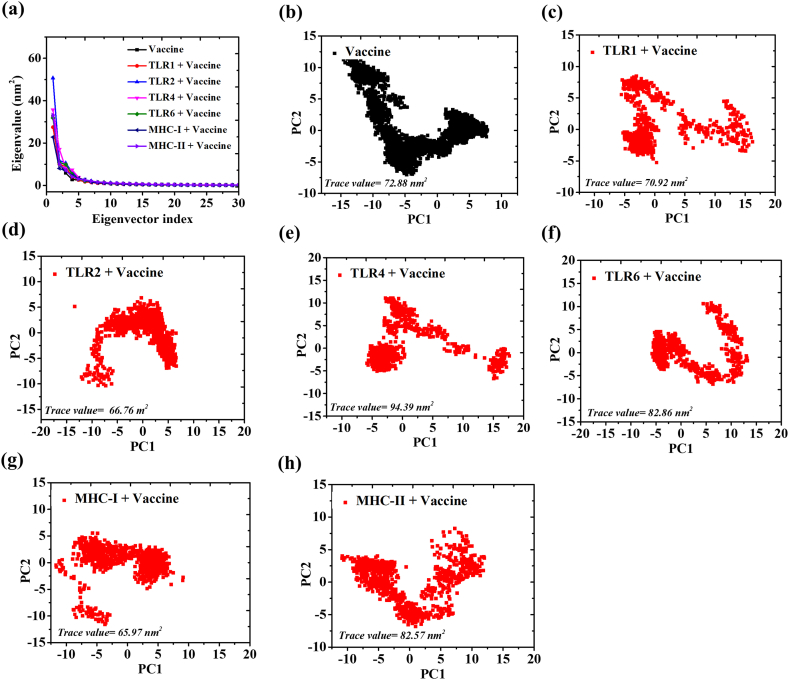
PCA analysis. The displacement of Cα atoms along the eigenvector 1 and 2 for all systems are shown in panel a. The 2D representation of motion, based on the first two eigenvectors obtained through principal component analysis (PCA), is depicted for Vaccine only and the Vaccine with TLRs and MHC receptors in panel b–h.

Principal component analysis (PCA) is a multivariate technique used to extract most significant modes of motion of protein system in MD simulation [20]. PCA was conducted on all seven systems comprising vaccine and vaccine-immune receptor complexes to gain understanding of the correlated motions of the proteins into principal motion which is characterized by an eigenvector and eigenvalues (Fig. 10). The first two eigenvectors delineate a crucial conformational subspace marked by substantial concerted motions, accounting for approximately 42 % in Vaccine, 38 % in TLR1+Vaccine, 62 % in TLR2+Vaccine, 53 % in TLR4+Vaccine, 43 % in TLR6+Vaccine, 31 % in MHC-I, and 43 % in MHC-II. These vectors were considered for the analysis of conformational dynamics (Fig. 10a). Further, overall flexibility of MD simulation systems were analysed by trace value (Fig. 10a–g). The TLR4+Vaccine exhibit maximum trace value (T.V. = 94.39 nm^2^) followed by TLR6+Vaccine (T.V. = 82.86 nm^2^), MHC-II + Vaccine (T.V. = 82.57 nm^2^), TLR1+Vaccine (T.V. = 70.92 nm^2^), TLR2+Vaccine (T.V. = 66.76 nm^2^) and MHC-I + Vaccine (T.V. = 65.97 nm^2^). TLR4 showed maximum trace value, which indicated that increase in the flexibility of vaccine on binding with human TLR4 receptor.

### 2.9 Computational cloning and immune simulation

**Fig. 11:**
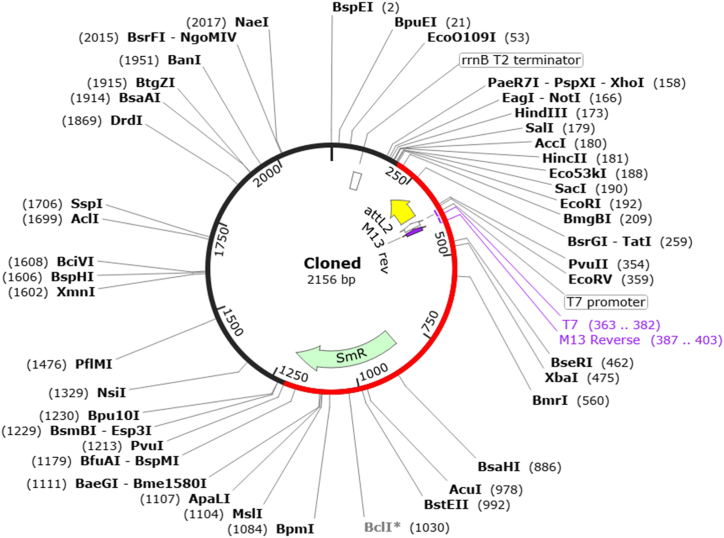
*In silico* cloning for expression of recombinant protein. The Mpox vaccine construct carrying multiple epitopes was virtually cloned into the pET-28b(+) expression vector, with the inserted segment depicted in red and the remaining sections representing the vector genome.

Computational cloning is important in the design and construction of vaccine constructs. We have converted the amino acid sequence of the designed vaccine into nucleotide by using EMBOSS Backtranseq server. The codon optimization resulted in a CAI score of 0.98 and a GC content of 54.80 %, suggested the promising potential of the designed vaccine for expression in the host cell (E. coli). In the next step, we choose the cloning vector, pET-28b(+), for the insertion and expression of vaccine construct. The generation in the pET-28b(+) vector was successfully done by using SnapGene tool (Fig. 11), indicated that vaccine construct can be easily expressed within the host cells.

**Fig. 12:**
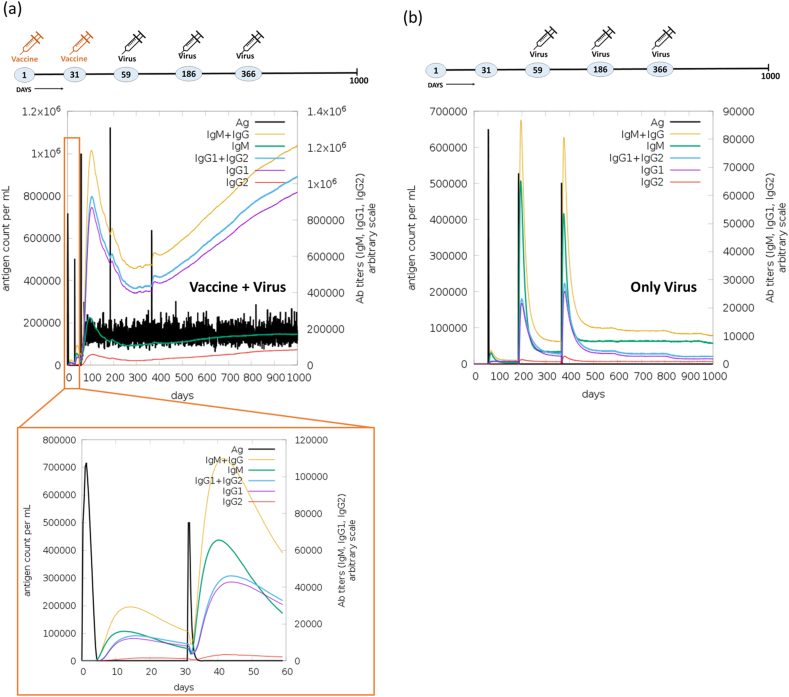
Comparative immune simulation analyses showing high immunogenicity of the multi-epitope Mpox vaccine. (a) Immune response of administration of Mpox vaccine on 1st and 31st day followed by addition of virus on 59th, 186th and 366th day. (b) Control experiment involving administration of only virus at on 59th, 186th and 366th day.

**Fig. 13:**
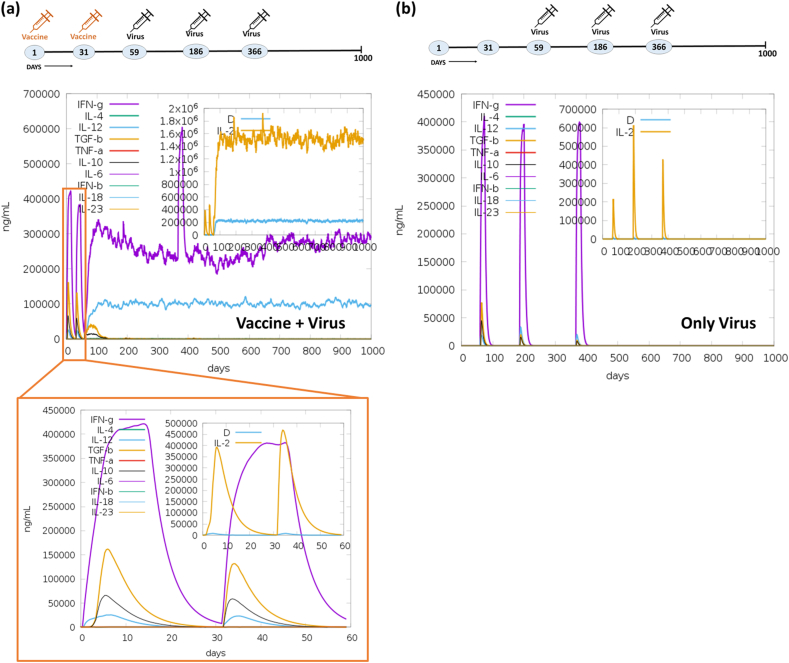
**Abundance of T-cells activating and proliferating cytokines in the immune simulation studies with Mpox vaccine.** Concentrations of cytokines and interleukins (ILs) were assessed in the comparative experiment involving (a) Vaccine + Virus and (b) Only Virus. The inset plot illustrates the presence of danger signals alongside the leukocyte growth factor IL2.

**Fig. 14:**
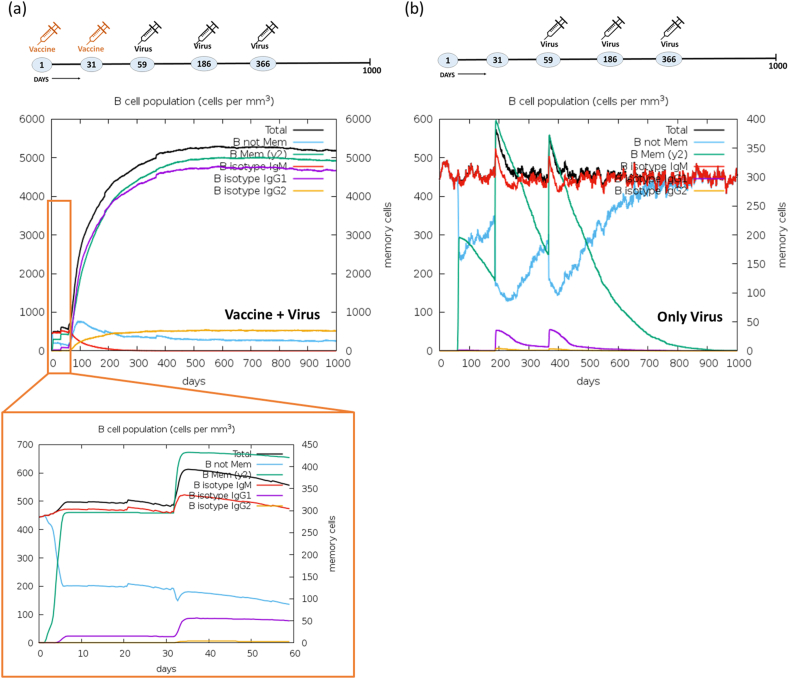
Activation and proliferation of B-cells. Activation of B-cells population (cells per mm^3^) in the comparative experiment of (a) Vaccine + Virus (b) Only Virus at different time points. Cell counts are shown in per mm^3^ human blood.

In immune simulation, two experiments were undertaken to assess the immune response elicited by designed vaccine when exposed to a virus. Initially, we immunized with the Mpox vaccine using online server (C-ImmSim), followed by one booster dose within a span of two months. Subsequently, we challenged with three doses of the virus to assess the immunogenicity of the vaccine. In the initial experiment, the vaccine was administered on the 1st and 31st day, and challenged with the Mpox virus on the 59th, 186th, and 366th day. In the second (control) experiment, only the Mpox virus was injected on the 59th, 186th, and 366th day to establish a basis for comparison with the immune response elicited by the vaccine. Notably, following the initial vaccine dose, immunoglobulin production was detected within five days. The activation of IgM + IgG, Ag, IgM, IgG1+IgG2, IgG1, and IgG2 was observed during both the primary and secondary immune responses, as illustrated in Fig. 12a. Markedly, the immune response demonstrated a five folds increase (1.1 × 10^5^) upon administration of the second vaccine dose. Upon challenging with the virus over three consecutive months (on the 59th, 186th and 366th day) after immunization with two vaccine dose, a substantial production of IgM + IgG amounting to 1.2 × 10^6^ was observed. This was succeeded by the continued production of IgM and IgG1, maintaining their activity up to the 1000th day. In the control experiment, the immune response exhibited a notably lower magnitude. Furthermore, the introduction of the vaccine on the 1st and 31st day leads to the production of cytokines, notably IFN-γ (>4 × 10^5^ ng/mL), TGF-β (>1.5 × 10^5^ ng/mL), IL10 (>6 × 10^4^ ng/mL), and IL12 (2.5 × 10^4^ ng/mL) (see Fig. 11a). Subsequently, upon administering three doses of the virus, an immediate IFN-γ response was observed. Remarkably, the continued activity of IFN-γ, IL12, and IL2 production was sustained for 1000 days, as depicted in Fig. 13a. Conversely, in the control experiment, the production of cytokines was not sustained for an extended duration, as illustrated in Fig. 13b. Moreover, the vaccine underwent testing for B-cells, T-cells, NK cells, dendritic cells (DC), macrophages (MA), and epithelial cells population (EP) immune responses. The concurrent administration of the vaccine and virus treatment resulted in a substantial memory B-cells response (∼5000 cells per mm^3^) and a significant augmentation of B-cells isotype IgG1 population (>4000 cells per mm^3^), as depicted in Fig. 14a. In contrast, the control experiment exhibited a weaker response of memory B-cell (maximum 600 cells per mm^3^) and a lower B-cells isotype IgM population (300 cells per mm^3^) as shown in Fig. 14b. Upon the administration of the vaccine on day 1 & 31, a notable peak in plasma B lymphocytes (PLB) occurred, specifically for IgM + IgG (∼100 cells/mm^3^), IgM (∼60 cells/mm^3^), and IgG1 (40 cells/mm^3^) immunoglobulins (Fig. S3a). Subsequently, this immune response was significantly amplified to approximately ∼275 cells/mm^3^ after the challenge with the live virus for three consecutive months. In contrast, the control experiment showed no significant response in the plasma B-lymphocytes (PLB) population (Fig. S3b).

**Fig. 15:**
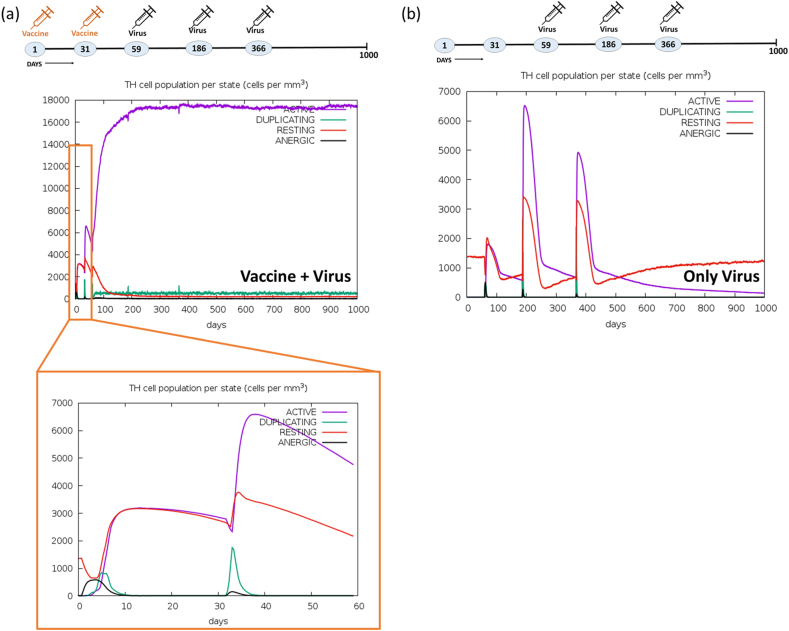
Activation and proliferation of Th-cells. Augmentation of CD4^+^ Th-cells population per state (cells per mm^3^) in the comparative experiment of (a) Vaccine + Virus (b) Only Virus at different time points. Cell counts are shown in per mm^3^ human blood.

**Fig. 16:**
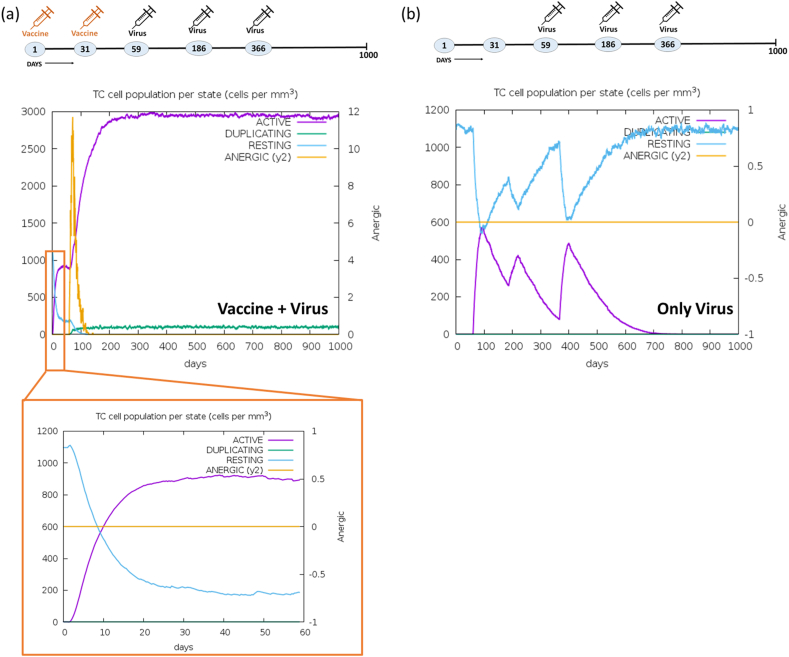
Activation and proliferation of Tc-cells. Concentration of CD8^+^ Tc-cells population per state (cells per mm^3^) in the comparative experiment of (a) Vaccine + Virus (b) Only Virus at different time points. Cell counts are shown in per mm^3^ human blood.

Moreover, the assessment of the CD4^+^ Th-cells population was conducted. The administration of two vaccine doses resulted in an approximate count of <6500 cells per mm^3^ within the active Th-cells population. Following infection with a third dose of the virus, this count increased significantly to 18000 cells per mm^3^, maintaining consistency over a period of 1000 days, as illustrated in Fig. 15a. In the control experiment, the duration of immune response upon virus injection was notably brief (Fig. 15b). Furthermore, the response of regulatory T-cells population (TR; 200 cells per mm^3^) were seen for only 100 days in the vaccine + virus experiment. Conversely, the immunological response of TR cells in the control experiment showed three distinct peaks on the day of injection. The CD8^+^ Tc-cells population, in response to a two-dose vaccine, surged to 800–1000 cells/mm^3^ within 15 days (Fig. 16a). Subsequently, this value of CD8^+^ Tc cell increased to 3000 cells/mm^3^ after the administration of three virus doses, maintaining consistency for an impressive duration of 1000 days, attesting to the robust immunogenicity of the vaccine. In contrast, the control experiment revealed fluctuating patterns in both the resting Tc-cells population (600–1200 cells/mm^3^) and the active Tc-cells population (0–600 cells/mm^3^) throughout the 1000-day observation period (Fig. 16b). No discernible disparities were noted in the populations of NK cells (Figs. S5a–b), DC cells (Figs. S6a–b), and macrophages (MA) (Figs. S6c–d) in both conditions: vaccine + virus & only virus. Within the vaccine environment, an active and actively infected epithelial cells (EP) population of 400 cells/mm^3^ was evident, but this diminished to 100–150 cells/mm^3^ in the virus-treated experiment (Fig. S7a). In contrast, the control experiment exhibited a singular curve depicting the active EP population at 400 cells/mm^3^ (Fig. S7b). In conclusion, the vaccine formulation elicited an increase in immunoglobulins, cytokines, B-cells, PLB cells, CD4^+^ Th-cells, CD8^+^ Tc-cells, and the EP population. This enhancement can be attributed to the incorporation of multi-epitopes in the vaccine design, contributing significantly to the overall immunogenicity of the vaccine. To sum up, the results indicated that the multi-epitope hybrid elicited a robust immune response upon initial exposure, and repeated exposures subsequently intensified this immune response.

## 3 Discussion

Mpox, a zoonotic disease caused by the Mpox virus (MPXV), has emerged as a significant public health concern due to its capacity for human-to-human transmission and the absence of its specific vaccine. The comprehensive immune response generated by multi-epitope vaccines offers increased effectiveness in the face of mutations. By encompassing epitopes from diverse viral proteins, these vaccines enhance their chances of remaining efficacious even if mutations affect specific epitopes. Inclusion of epitopes from conserved regions across different viral proteins broadens the protective scope, as these regions are less susceptible to mutations compared to variable regions, ensuring prolonged immune effectiveness. The inherent complexity of multi-epitope vaccines diminishes the likelihood of escape mutants emerging, requiring the virus to undergo simultaneous mutations in multiple epitopes, a less probable scenario. The development of a cross-reactive immune response by targeting various proteins ensures continued protection, even in the presence of significant mutations in one protein. The concurrent targeting of multiple epitopes exerts mutational pressure on the virus, heightening the challenge for the virus to evade the immune response. Immunoinformatics has brought about a revolutionary shift in immunology research and holds the promise of transforming the domains of vaccine development and immunotherapy. Using that platform, we have developed a multi-epitope hybrid vaccine against the Mpox virus. Epitope-based vaccines can be developed by identifying the most immunogenic epitopes and formulating them in a way that enhances their stability and presentation to the immune system. There are primarily two types of immune cell epitopes that are important in vaccine development: B-cell epitopes and T-cell epitopes. We have identified B-cell epitopes (size ≥8aa), in which 1 from A30L, 4 from L1R, 4 from M1R and 3 from E8L glycoproteins of Mpox virus. These B-cell epitopes will facilitate immune recognition and antibody production, leading to the production of antibodies that bind to pathogens, neutralize them, and enhance the immune response. The T-cell epitopes includes 4 Tc-cells (size = 9aa) and 3 Th-cells (size = 15aa) were selected from A30L, A35R and E8L glycoproteins. These predicted T-cell epitopes will drive cellular immune responses and enhance immune memory, resulting in the activation of T cells, clearance of intracellular pathogens, and the growth of long-term immunity. A total of five IFN-γ epitopes, comprising one epitope (size = 15aa) each from the A29L, A30L, A35R, L1R, and M1R glycoproteins of the Mpox virus, were predicted for the vaccine design. These epitopes will expected to stimulate the generation of IFN-γ, a key cytokine that activates immune cells, enhances antigen presentation, and promotes effective robust immune response against pathogens. These selected epitopes for the vaccine construct exhibit extensive coverage across HLA populations worldwide. HLA molecules play a crucial role in presenting epitopes to the immune system, and the diversity of HLA alleles across populations can impact vaccine efficacy. Therefore, assessing the HLA population coverage of epitopes helps determine the potential effectiveness of a vaccine in eliciting immune responses in various populations globally in combating diseases like Monkeypox. To improve the immune response of the vaccine, PADRE peptide is used an adjuvant in the vaccine construct. It offers valuable advantages in vaccine development, including its immunostimulatory properties, ability to activate helper T cells, improved antigen presentation, enhanced immunogenicity, and proven safety. In order to enhance the structural integrity of the vaccine and the optimization of antigen processing, different linkers (EAAAK, GPGPG, and AAY) were employed to connect the T-cell, B-cell, and IFN-γ epitopes in the final vaccine framework. We selected the EAAAK linker for its ability to form a stable alpha-helix structure. The GPGPG linker is preferred in multi-epitope vaccines due to its flexibility and non-immunogenic properties. It effectively separates the epitopes, minimizing unwanted immune responses at the junctions and enabling each epitope to be independently processed and presented. The AAY linker was chosen for its proficiency in aiding the proteasome to generate appropriate peptide fragments, which is crucial for presenting epitopes through the MHC class I pathway. PADRE, a universal helper T-cell epitope, is included to provide broad coverage across various human leukocyte antigen (HLA) haplotypes, ensuring a robust helper T-cell response. The vaccine demonstrates good antigenicity score and lacks allergic characteristics, indicating its potential as an immunogenic vaccine. The vaccine exhibits favorable physicochemical properties, stability, and a well-defined 3D structure, validated through homology modeling and refinement processes. MD simulations (RMSD, *R*_g_, RMSF, SASA, PCA and hydrogen bond analysis) provide understanding of the stability, flexibility, and interactions of the vaccine with receptors. The vaccine demonstrates strong binding affinity with TLRs (TLR1, TLR4, TLR6) and MHC receptors (MHC-I, MHC-II), leading to the activation of immune responses. Predominantly B-cell epitopes and fewer IFN-γ and Th-cells epitopes of vaccine showed molecular interaction with TLR and MHC's receptor through hydrogen bonding and hydrophobic interaction. The GC content (54.80 %) and CAI score (0.98) validated the high expression of the designed vaccines in the pET-28b (+) expression vector. Furthermore, two additional experiments were carried out to assess the immune response elicited by a formulated vaccine using immune simulation. In the initial experiment, the immune system was primed with a single vaccine dose, followed by a booster dose after a significant time interval to induce a memory response through germinal center activation. This was then challenged with three doses of live virus. In contrast, the control experiment received only virus doses for comparative analysis. *In silico* immune simulations estimated the vaccine's potential to activate protective immunity, demonstrating its capacity to generate a robust immune memory response capable of countering live virus challenges. As anticipated, it was demonstrated through the production of immunoglobulins (IgM + IgG, IgM, IgG1+IgG2), cytokine release (IFN-γ, IL10, IL12, and TGF-β), and the activation of various immune memory responses such as T memory and B memory. Thorough validation of the formulated vaccine through diverse parameters affirms its capability to tackle challenges related to infectious diseases caused by mutated virus, leading to enhanced public health outcomes. The vaccine provides a wider range of protection, heightened efficacy, and cost-effective measures for disease prevention and control.

## 4 Conclusions

The present study successfully identified and evaluated six glycoproteins from the Mpox virus to construct a multi-epitope hybrid vaccine. The chosen epitopes demonstrated strong antigenicity and immunogenicity, and the vaccine was assembled with these epitopes, an adjuvant, and specific linkers. The physicochemical properties, 3D structure, and stability of the vaccine were thoroughly analysed. Molecular docking and dynamics simulations confirmed effective interactions between the vaccine and immune receptors, with significant contributions from B-cell and IFN-γ specific epitopes. Immune simulations showed that the vaccine elicited a robust immune response, producing antibodies and activating various immune cells, with an enhanced immune memory response following a booster dose. The study highlights the potential of immunoinformatics in vaccine development, providing promising insights into a multi-epitope Mpox vaccine.

## 5 Methodology

### 5.1 Sequence retrieval of glycoproteins and its immunogenic peptide prediction

**Fig. 17:**
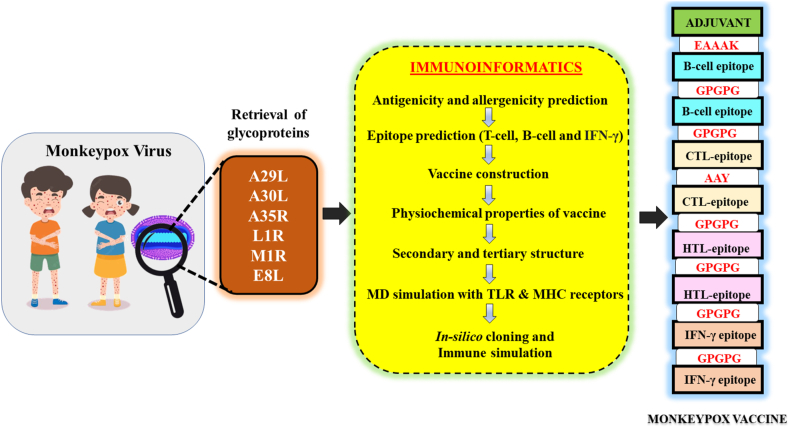
The workflow of development of multi-epitope monkeypox vaccine. Strategic flowchart of developing a multi-epitope monkeypox vaccine, showcasing the systematic design and construction process to enhance efficacy and immune response.

The NCBI website was employed to extract the sequence of the six glycoproteins (A29L, A30L, A35R, L1R, M1R, and E8L) of the Mpox virus. The antigenicity and allergenicity of all the glycoproteins were estimated using VaxiJen v2.0 server [21] and AllerTop v. 2.0 [22], respectively. Proteins having an antigenicity score of less than 0.4 are regarded as antigens, which is pivotal in triggering immunity to the Mpox virus. Fig. 17 depicts the overall procedure for developing a multiepitope vaccination that selectively targets the Mpox virus.

### 5.2 Physiochemical properties and secondary structure glycoproteins

The physiochemical characteristics of all glycoproteins, such as MW, isoelectric point, instability index, and aliphatic index, were examined using the web tool Expasy protpram [23]. Further, the secondary structural elements such as α-helix, 3_10_ helix, π-helix, β bridge, extended strand, β turn, β region, and a random coil of each glycoprotein were analysed by SOPMA online tool [24].

### 5.3 Prediction of Tc-cell) and Th-cells specific epitopes

All glycoproteins were subjected to NetCTL.1.2 server for the prediction of Tc-cells epitopes. The peptides which were recognized by different types of HLA supertypes (A1, A2, A3, A24, A26, B7, B8, B27, B39, B44, B58, B62) were selected based on their threshold values. Another group of Tc-cells epitopes was scrutinized by Immune Epitope Database (IEDB) tool using ANN method and screened based on their IC50 value (≤100) [25]. The epitopes that are associated multiple alleles are believed to be effective binders, so they were chosen for further investigation. Further, Net MHC II pan 3.2 server is used to identify Th-cells epitopes recognized by various HLA Class II DR alleles (HLA-DRB1*01:01, HLA-DRB1*03:01, HLA-DRB1*04:01, HLA-DRB1*04:05, HLA-DRB1*07:01, HLA-DRB1*08:02, HLA-DRB1*09:01, HLA-DRB1*11:01, HLA-DRB1*12:01, HLA-DRB1*13:02, HLA-DRB1*15:01, HLA-DRB3*01:01, HLA-DRB3*02:02, HLA-DRB4*01:01, HLA-DRB5*01:01, HLA-DQA1*05:01/DQB1*02:01, HLA-DQA1*05:01/DQB1*03:01, HLA-DQA1*03:01/DQB1*03:02, HLA-DQA1*04:01/DQB1*04:02, HLA-DQA1*01:01/DQB1*05:01, HLA-DQA1*01:02/DQB1*06:02, HLA-DPA1*02:01/DPB1*01:01, HLA-DPA1*01:03/DPB1*02:01, HLA-DPA1*01:03/DPB1*04:01, HLA-DPA1*03:01/DPB1*04:02, HLA-DPA1*02:01/DPB1*05:01, HLA-DPA1*02:01/DPB1*14:01).

### 5.4 Scrutinized overlapping T cell epitopes

Epitopes with an affinity for many HLA alleles are likely to generate a greater immunological response within the host cell. Therefore, we have overlay Tc-cells and Th-cells epitopes, and selected those epitopes which showed binding towards HLA class I and II alleles. Further, the selection of the overlapped epitopes were on their antigenicity score (VaxiJen v2.0) and allerginicity (AllerTop v2.0). Such epitopes were thought to have a high potential for triggering T-cells.

### 5.5 Prediction of B-cells and IFN-γ epitopes

Memory cells and plasma cells are two types of B-lymphocytes (B-cell) that release antibodies on the membrane in response to antigens. There are two types of B-cell epitopes: continuous (Table S2) and discontinuous which plays a significant role in vaccine development. We have identified linear B-cell epitopes for all glycoproteins using IEDB tool (http://tools.iedb.org/bcell/) [26]. Similarly, ElliPro: Antibody Epitope Prediction tool was used to predict discontinuous B-cell epitopes with score higher than 0.8 [27]. IFN-γ also known as type II IFN, is a type of cytokine that is essential for natural and acquired immunity against bacteria, viruses, and protozoan infections. Thus, IFN-γ-inducing epitopes could improve the immunogenicity of any vaccination. The “IFNepitope” online tool is employed to predict IFN-γ epitopes from all eight glycoproteins by using Motif and SVM hybrid approaches [28].

### 5.6 Conservation and population coverage analysis

The IEDB conservancy analysis tool is utilized to examine the extent of conservation of the chosen epitopes in all glycoproteins [29]. IEDB provides another tool “Population coverage”, which can be used for peptide-based vaccines and diagnostics. The selected sequence of Tc-cells and Th-cells epitopes have been uploaded to the IEDB tool, only those with 100 % conservancy were chosen for vaccine construction.

### 5.7 Construction, characterization and modelling of vaccine

All Tc-cells, Th-cells, B-cell and IFN-γ epitopes chosen for the construction of multi-epitope hybrid vaccine, were antigenic, non-allergen, showed strong binding affinity towards both MHC-I and MHC-II alleles and possessed more than 50 % population coverage. The PADRE peptide (AKFVAAWTLKAAA) is used as an adjuvant positioned at the N-terminus of the vaccine to activate particular CD4^+^ T-cells while leading to an innate immunological response. To attach the adjuvant to the B-cell epitopes, an EAAAK peptide was used. GPGPG peptide is used to link B-cell with Tc-cells, Tc-cells with Th-cells and Th-cells with IFN-γ epitopes for the vaccine assembly. Tc-cells epitopes are linked together using the AAY peptide, whereas Th-cells, B-cells, and IFN-γ epitopes are joined using the GPGPG peptide linker.

The antigenicity and allergenicity of the vaccine were estimated by VaxiJen v2.0 webserver and AllerTop v. 2.0. The chemical and physical attributes of the vaccine were studied using Expasy protpram. SOPMA webserver, was used to predict the secondary structure of the vaccine. To obtain the 3D model of vaccine, the sequence of vaccine was submitted to three different homology servers: (i) I-tasser, (ii) Robetta and (iii) IntFOLD. For refinement, the structures obtained from three servers were submitted to Galaxy refine server. The conformational quality of obtained 3D structures of vaccine after refinement was analysed by Ramachandran plot using MolProbity server. The structure with the least Rama outliers % and highest Rama favoured % is chosen for molecular docking and simulations studies. The 3D structure was visualized by PyMOL visualization system.

### 5.8 Molecular docking and molecular dynamics (MD) simulation

The 3D structures of TLR1, TLR2, TLR4, TLR6, MHC class I and MHC class II were obtained from Protein Data Bank having PDB ID: 2Z7X, 2Z7X, 2Z63, 3A79, 3OX8 and 2IPK, respectively. The binding efficacy of the vaccine with various TLR receptors (TLR1, TLR2, TLR4 and TLR6) and MHC class I & II was studied using the ClusPro server [30]. The best-docked pose with the lowest energy weight was chosen for the binding interaction study. The binding energy and K_d_ between protein-protein was calculated by PRODIGY webserver [31]. PyMOL and LigPlot^+^software was used to visualise the hydrogen bonding and hydrophobic interaction between vaccine and receptor [32,33]. The best-docked structure obtained were later used as the initial structure for the MD simulation by using GROMACS package [34]. Total seven systems were prepared for the simulation; (i) Vaccine, (ii) TLR1 + Vaccine, (iii) TLR2 + Vaccine, (iv) TLR4 + Vaccine, (v) TLR6 + Vaccine, (vi) MHC-I + Vaccine and (vii) MHC-II + Vaccine. Simulation (i) was run for 300 ns whereas simulation (ii-vii) were run for 50 ns each.

The two additional independent simulations of vaccine were performed for 100 ns and one additional simulation of 50 ns each were performed for all vaccine-receptor simulations. The topology of all protein complexes in explicit solvent was generated using the AMBER99SB-ILDN force field using “tip3p” water model [35,36]. We have performed 50000 steps for the steepest descent energy minimization of all systems. The simulation employed a Verlet cutoff scheme along with the particle-mesh Ewald (PME) method to calculate long-range electrostatic interactions [37,38]. The pressure and temperature were kept at 1.0 bar and 310 K using the Parrinello−Rahman barostat and modified Berendsen thermostat, respectively, for the MD simulation [39,40]. Lincs constraint algorithm was used for constraining the atoms [41]. The graphs were generated using OriginPro 9.0 software and molecular structures were visualized by PyMOL software. The MD trajectories were analysed using GROMACS tools. The conformational stability of proteins in the absence or presence of ligands was analysed using gmx rms, gmx rmsf, gmx gyrate and gmx sasa tools. The Daura et al. algorithm was utilized to cluster MD simulation trajectories [42].

### 5.9 PCA analysis

To comprehend the correlated movement of a protein along its principal trajectory while occurring in a complex with another protein, we have employed Principal Component Analysis (PCA), a technique characterized by eigenvectors and eigenvalues [43,44]. Each eigenvector serves as a predictor of a specific direction, while the associated eigenvalue quantifies the magnitude of the corresponding motion. The primary eigenvector (PC1) indicates the direction in which the sample conformations exhibit the most significant variation. Simultaneously, the secondary eigenvector (PC2) represents a direction uncorrelated and orthogonal to PC1, showcasing the highest variation in sample conformations. Therefore, we have calculated first two principal component PC1 and PC2 for all the seven MD simulation system by using gmx in-built tool “gmx covar”.

### 5.10 In silico cloning, codon optimization and immune simulation

The efficacy of designed vaccine in cloning and expression is crucial for any vaccine design. We have used EMBOSS Backtranseq server to convert vaccine protein sequence to nucleotide sequence [45]. The CodonAdaptionTool (JCAT) was employed to optimize the codons of the vaccine construct in E. coli strain K12 to produce optimized vaccine sequence [46]. The CAI value score (>0.8) and GC content (30–70 %) indicated the post-translation, stability and transcription ability. The E. coli pET–28b(+) vector was used to clone the designed vaccine construct by using SnapGene 4.2 tool [47]. The immune simulation was conducted using C-ImmSim server to evaluate the immunogenic response of the designed multi-epitope Mpox vaccine [48].

## CRediT authorship contribution statement

**Anupamjeet Kaur:** Conceptualization, Data curation, Formal analysis, Investigation, Methodology, Software, Writing – original draft. **Amit Kumar:** Data curation, Formal analysis, Supervision, Validation. **Geetika Kumari:** Data curation, Formal analysis, Investigation, Methodology, Validation. **Rasmiranjan Muduli:** Data curation, Formal analysis, Investigation, Methodology. **Mayami Das:** Data curation, Formal analysis, Investigation, Methodology. **Rakesh Kundu:** Data curation, Formal analysis, Methodology, Supervision, Validation, Visualization. **Suprabhat Mukherjee:** Software, Supervision, Validation, Visualization. **Tanmay Majumdar:** Data curation, Methodology, Writing – original draft, Writing – review & editing.

## Declaration of competing interest

Authors declare that they have no competing interests.
