# Supplementary material for: Rational design and computational evaluation of a multi-epitope vaccine for monkeypox virus: Insights into binding stability and immunological memory
Source: Heliyon. 2024 Aug 20;10(16):e36154. doi: 10.1016/j.heliyon.2024.e36154 (PMC11380015; doi:10.1016/j.heliyon.2024.e36154)
Supplement: Multimedia component 1 [file mmc1.docx]

**Rational design and computational evaluation of a multi-epitope vaccine for Monkeypox Virus: Insights into binding stability and immunological memory**

**Supporting Information**

**
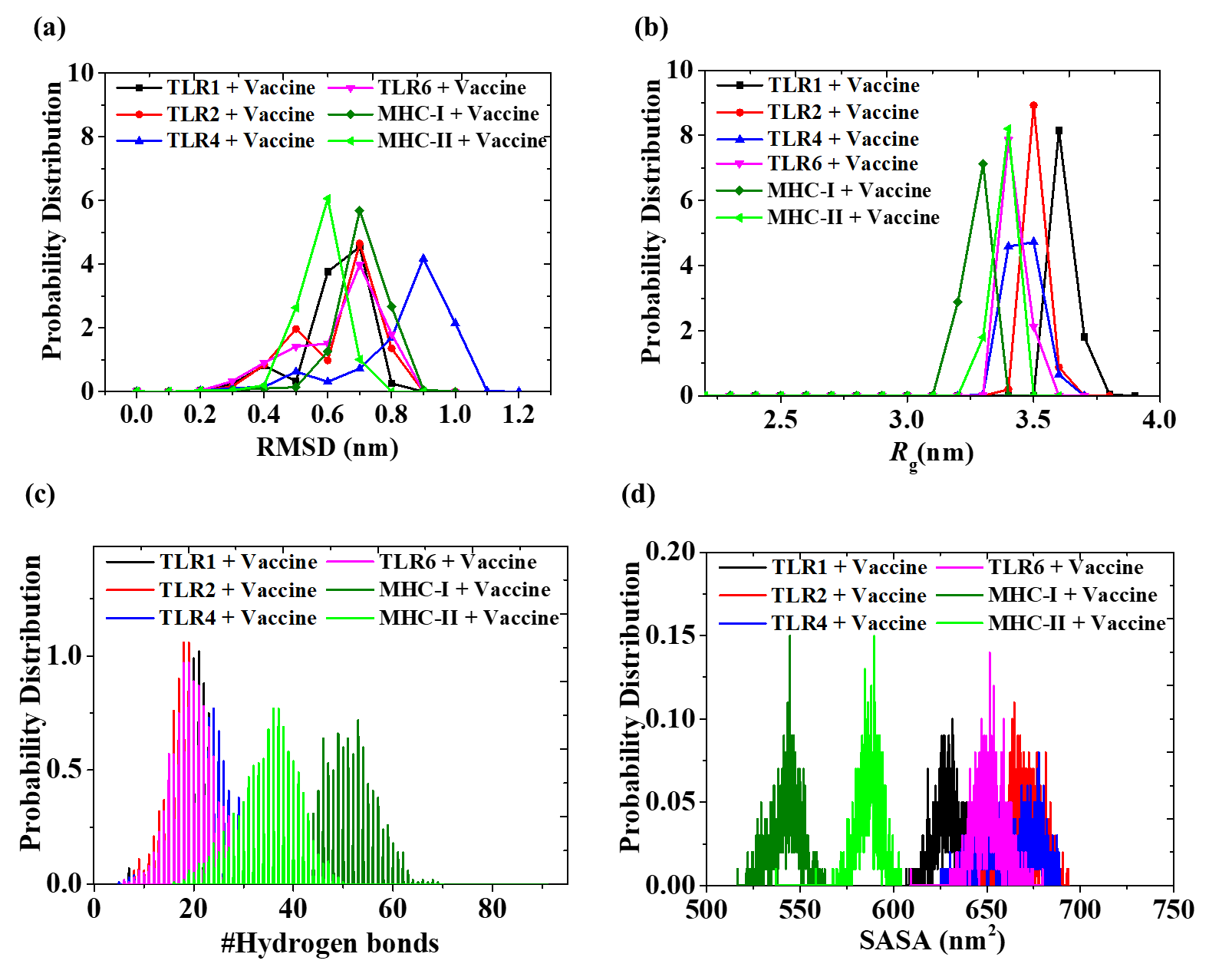
**

**Figure S1.** **Probability distribution graph** **of MD simulation of Mpox vaccine with TLR1, TLR2, TLR4, TLR6, MHC-I and MHC-II.** (a) RMSD (b) *R*_g_ (c) Number of hydrogen bond between Mpox vaccine and various receptors (TLR1, TLR2, TLR4, TLR6, MHC-I and MHC-II) (d) SASA .


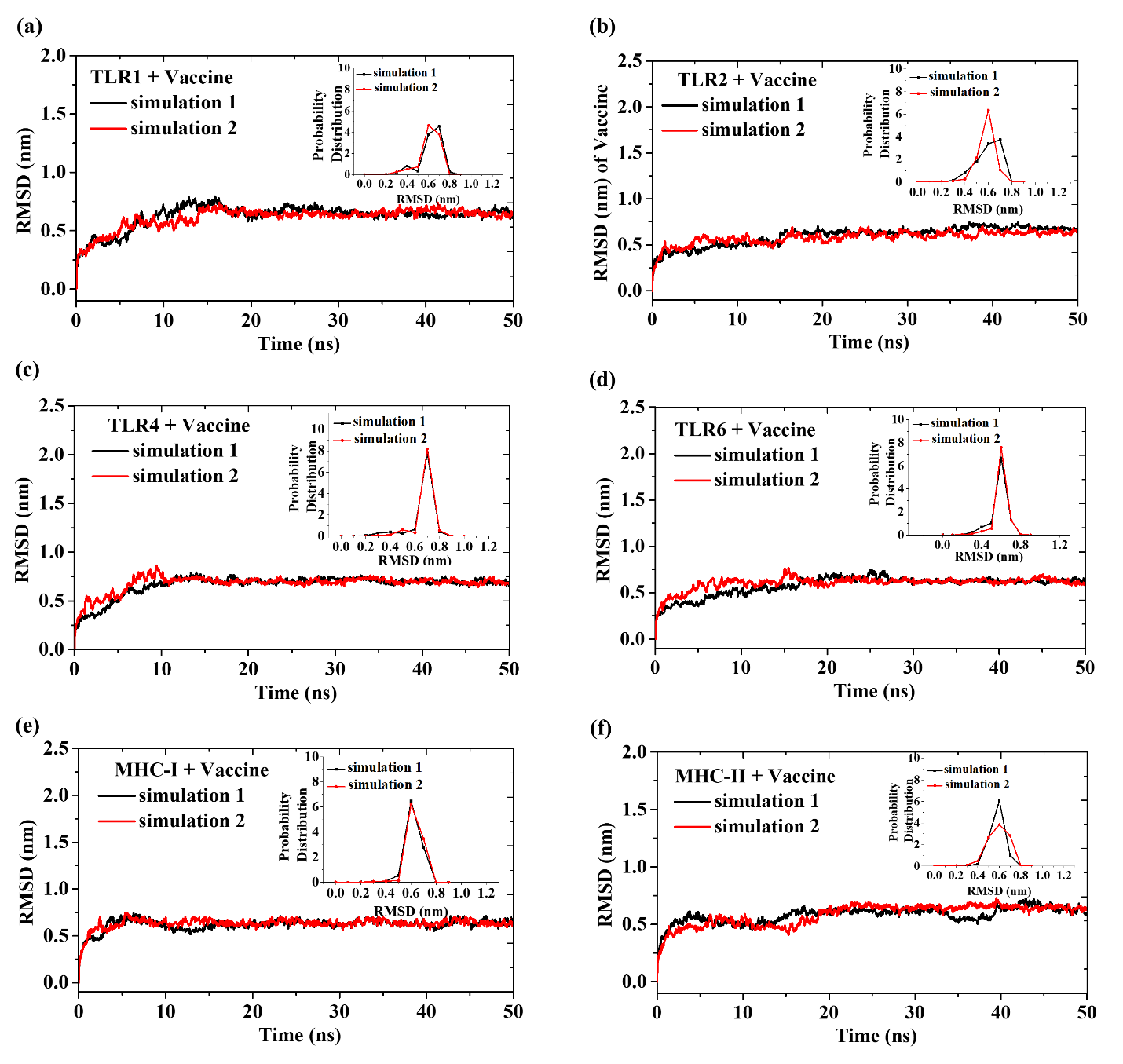


**Figure S2. MD simulations** **verifying vaccine-TLR interactions.** The RMSD of duplicate simulations for (a) TLR1 + Vaccine; (b) TLR2 + Vaccine; (c) TLR4 + Vaccine; (d) TLR6 + Vaccine; (e) MHC-I + Vaccine; and (f) MHC-II + Vaccine.


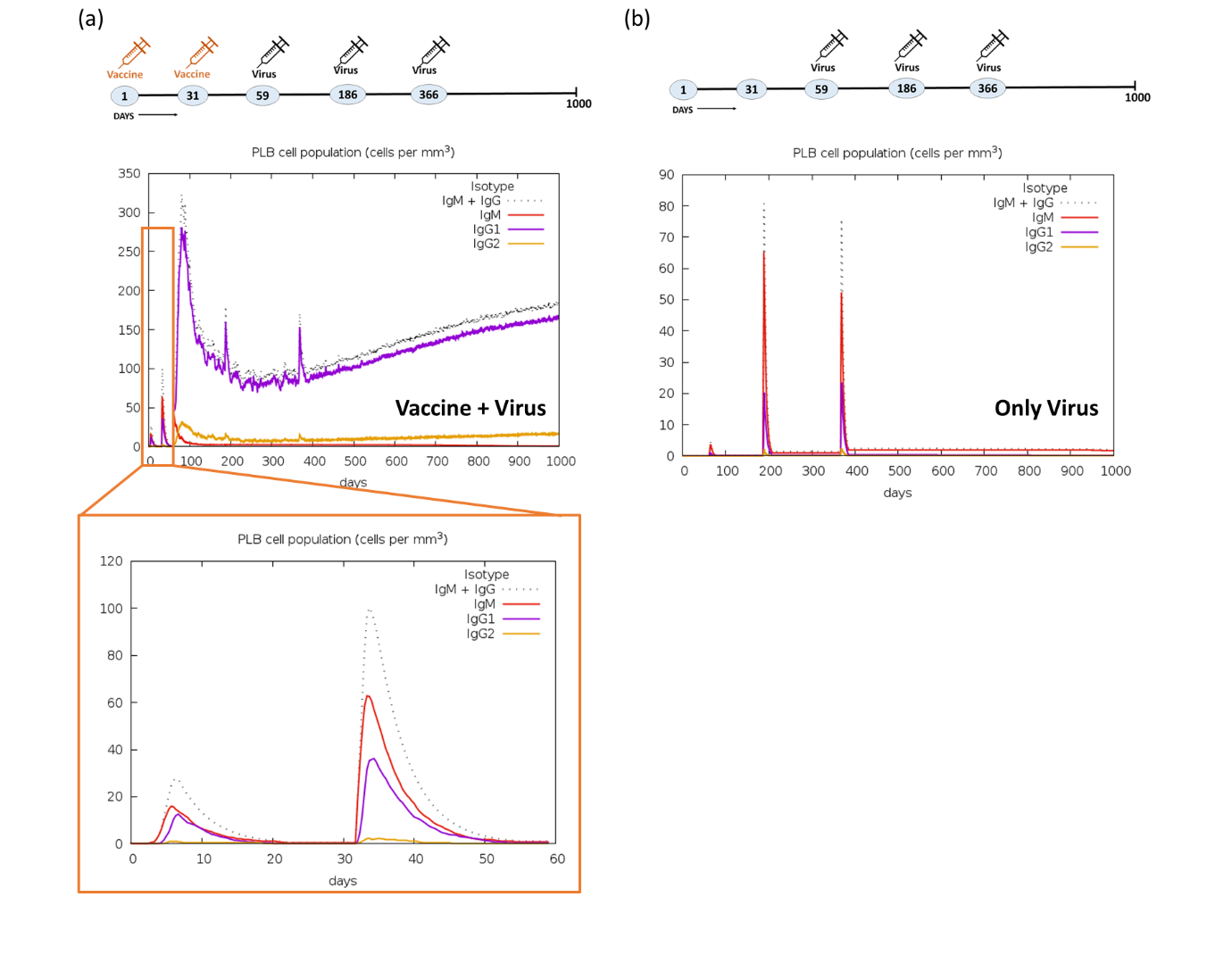


**Figure S3.** **Activation and proliferation of PLB-cells** **in response to the designed peptide vaccine.** Concentration of PLB population (cells per mm^3^) in the comparative experiment of (a) Vaccine + Virus (b) Only Virus at different time points. Cell counts shown.


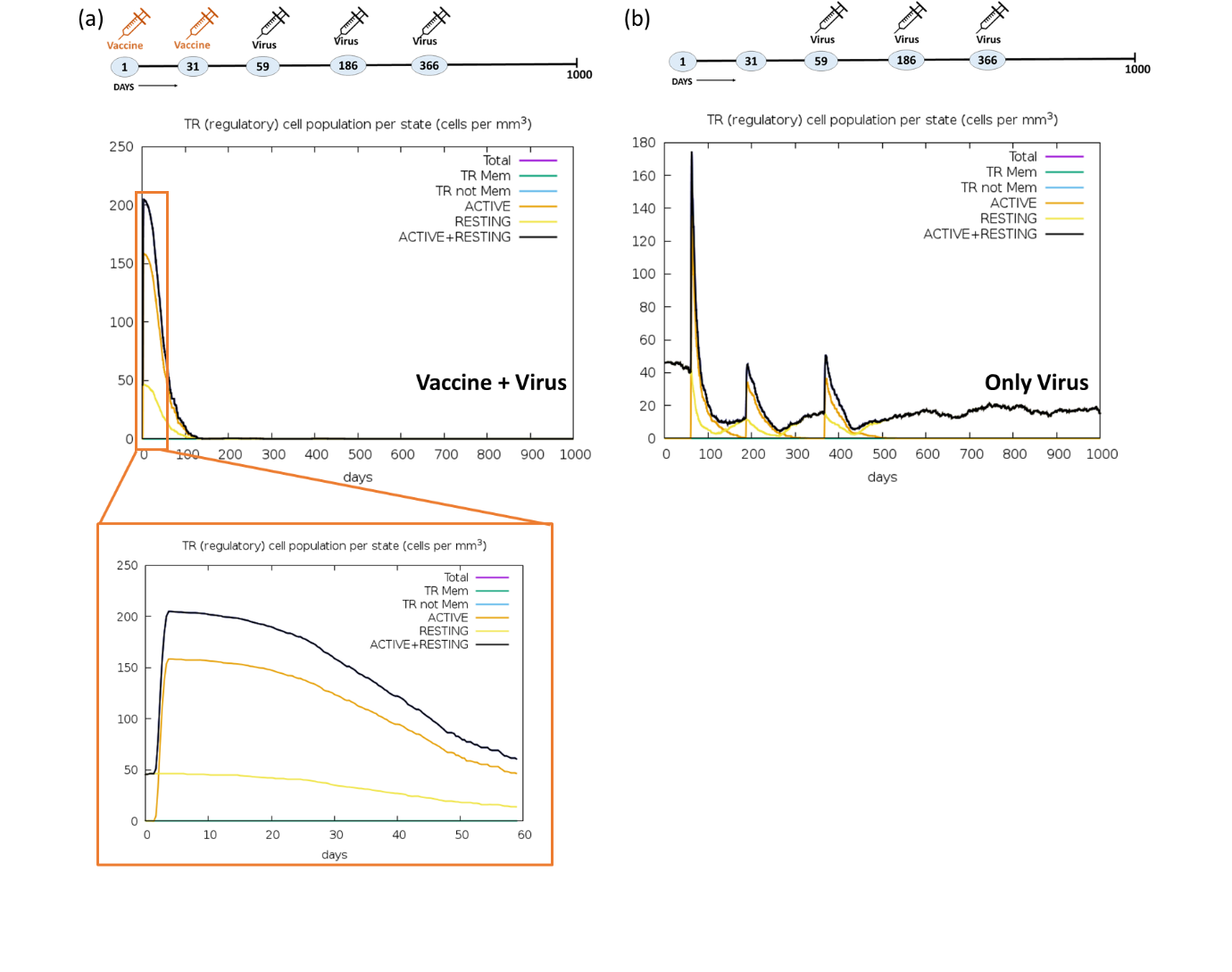


**Figure S4.** **Vaccine-induced** **activation and proliferation of TR cells.** Concentration of TR cell population per state (cells per mm^3^) in the comparative experiment of (a) Vaccine + Virus (b) Only Virus at different time points. Cell counts shown.


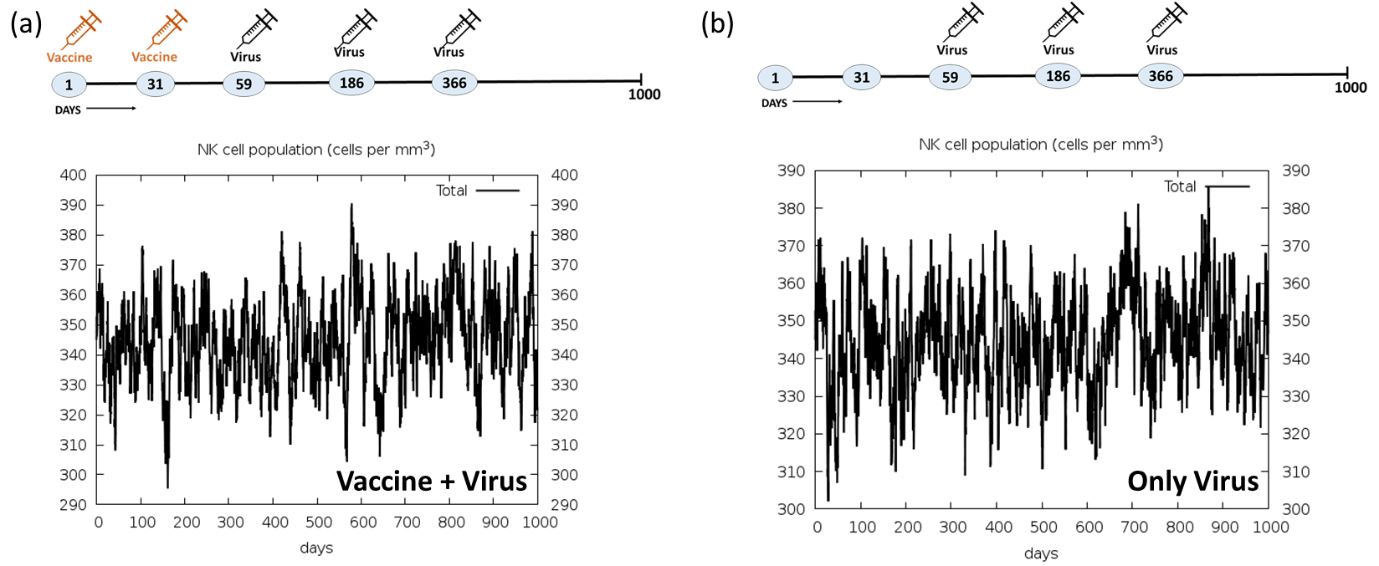


**Figure S5.** **Vaccine-induced activation and proliferation of NK cells** Concentration of NK cell population per state (cells per mm^3^) in the comparative experiment of (a) Vaccine + Virus (b) Only Virus at different time points. Cell counts shown.

**
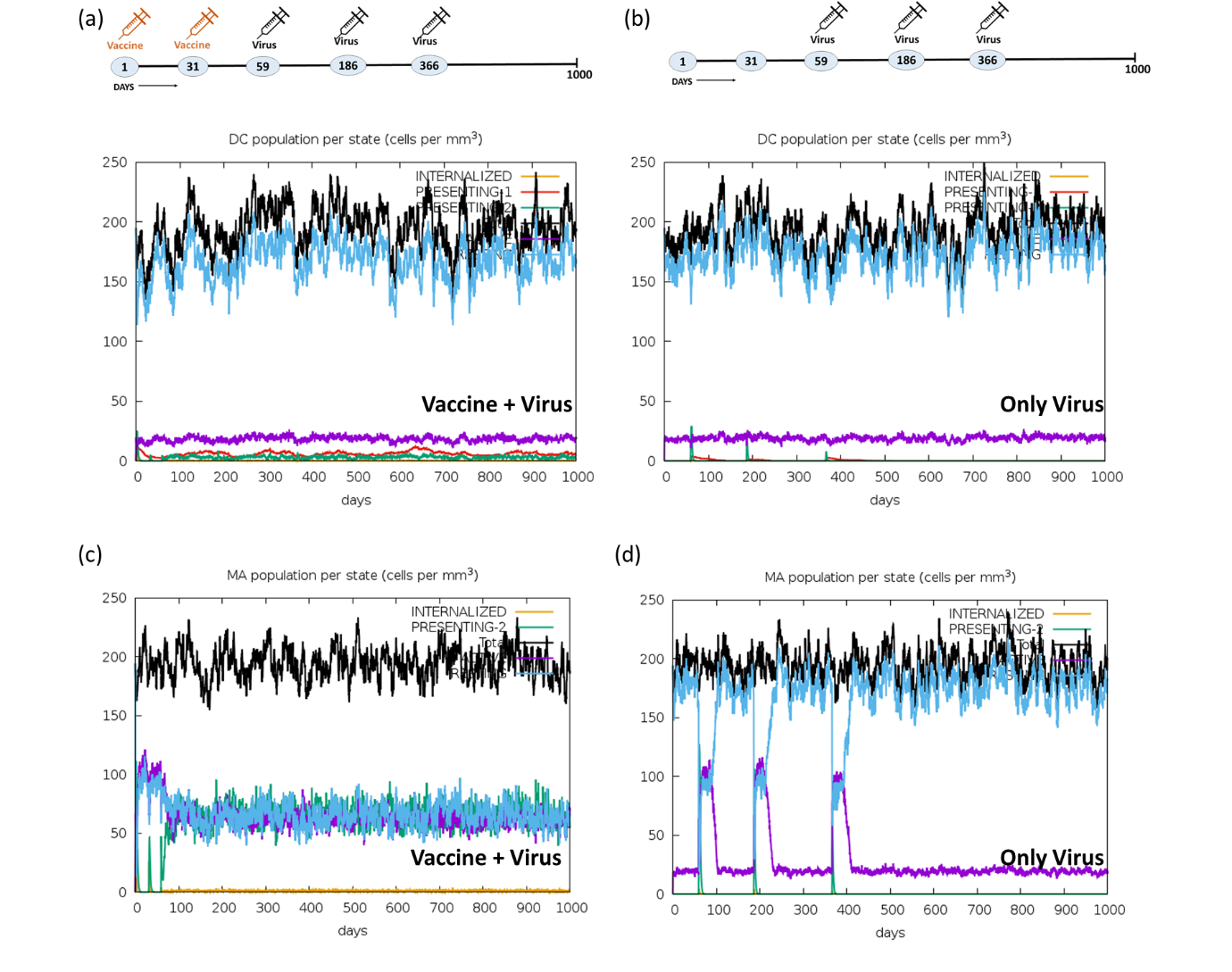
**

**Figure S6.** **Vaccine-induced activation and proliferation of DC, MA cells.** Concentration of DC cell population per state (cells per mm^3^) in the comparative experiment of (a) Vaccine + Virus (b) Only Virus at different time points. Concentration of MA cell population per state (cells per mm^3^) in the comparative experiment of (c) Vaccine + Virus (d) Only Virus at different time points. Cell counts shown.


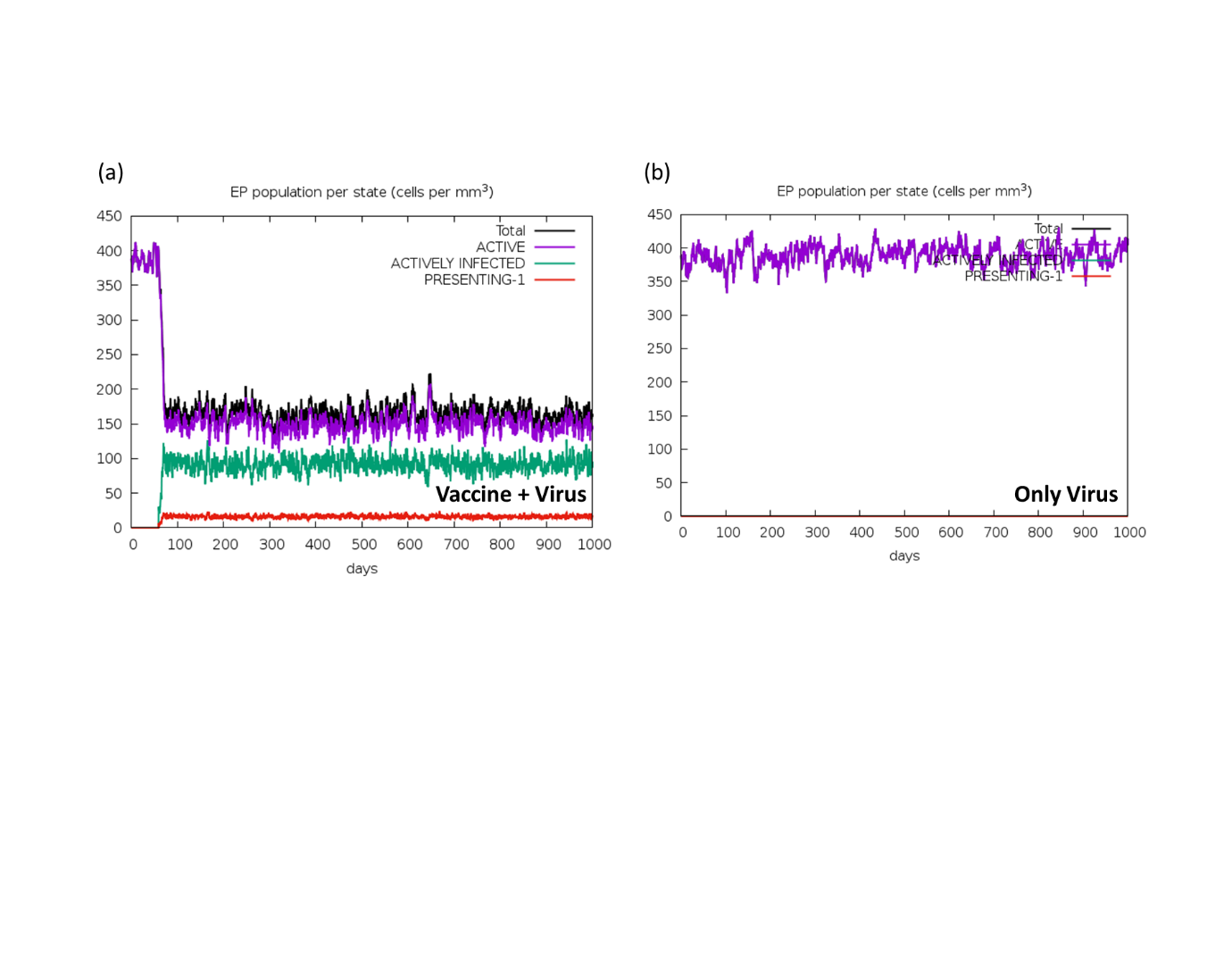


**Figure S7.** **Vaccine-induced activation and proliferation of EP cells.** Concentration of EP cell population per state (cells per mm^3^) in the comparative experiment of (a) Vaccine + Virus (b) Only Virus at different time points. Cell counts shown.

**Table S1**: Catalog of bioinformatics databases and tools utilized in this study.

| **Objective** | **Tools** | **URL** |
| --- | --- | --- |
| Sequence retrieval and analysis | NCBI database | <https://www.ncbi.nlm.nih.gov> |
| Antigenicity Assessment | VaxiJen | <http://www.ddg-pharmfac.net/vaxijen/VaxiJen/VaxiJen.html> |
| Allergenicity Assessment | AllerTOP | <https://www.ddg-pharmfac.net/AllerTOP/> |
| Physicochemical  Characterization | ProtParam | <http://web.expasy.org/protparam/> |
| Secondary Structure Characterisation | SOPMA  PSIPRED | <https://npsa.lyon.inserm.fr/cgi-bin/npsa_automat.pl?page=/NPSA/npsa_sopma.html>  <http://bioinf.cs.ucl.ac.uk/psipred/> |
| T-cell Epitope prediction | NetCTL 1.2  IEDB  NetMHCIIpan - 3.2 | <https://services.healthtech.dtu.dk/service.php?NetCTL-1.2>  <http://tools.iedb.org/mhci/>  <http://tools.iedb.org/mhcii/>  <https://services.healthtech.dtu.dk/service.php?NetMHCIIpan-3.2> |
| IFN-γ epitope prediction | IFN epitope server | <https://webs.iiitd.edu.in/raghava/ifnepitope/predict.php> |
| Continuous B-cell epitope prediction | Antibody Epitope Prediction | <http://tools.iedb.org/bcell/> |
| Discontinuous B-cell epitope prediction | ElliPro: Antibody Epitope Prediction | <http://tools.iedb.org/ellipro/> |
| Toxicity Prediction | Toxinpred | <http://crdd.osdd.net/raghava/toxinpred/> |
| Solubitlity prediction in *E. coli* | SoluProt | <https://loschmidt.chemi.muni.cz/soluprot/> |
| 3D structure prediction of Vaccine | I-Tasser  IntFOLD  Robetta | <https://zhanggroup.org/I-TASSER/>  <https://www.reading.ac.uk/bioinf/IntFOLD/>  <https://robetta.bakerlab.org/queue.php> |
| Refining of 3D structure | Galaxy refine | <https://galaxy.seoklab.org/cgi-bin/submit.cgi?type=REFINE> |
| Ramachandra Plot | MolProbity | <http://molprobity.biochem.duke.edu/> |
| Population Coverage | Population coverage | <http://tools.iedb.org/population/> |
| Molecular docking | Clupro 2.0 | <https://cluspro.org/help.php> |
| Molecular Dynamics | GROMACS package | <https://www.gromacs.org/> |
| Conversion of Protein sequence (vaccine) to Nucleotide sequence | EMBOSS Backtranseq | <https://www.ebi.ac.uk/Tools/st/emboss_backtranseq/> |
| Optimization of nucleotide sequence | Jcat CODON Adaptation tool | <http://www.jcat.de/> |
| *In silico* cloning of vaccine | Snapgene | <https://www.snapgene.com/> |
| Immune simulation | C-IMMSIM | <https://kraken.iac.rm.cnr.it/C-IMMSIM/> |

Table S2: Continuous B-cell epitopes identified using three distinct prediction tools

| **B-cell epitopes Prediction tool** | **Proteins** |
| --- | --- |
| **A29L** | |
| Ellipro  (from protein sequence) | MDGTLFPGDDDLAIPATEFFSTKAAKNPETKREAIVKAYGDDNEETLKITNITTKFEQIEKCCKRNDEVLFRLENHAETLRAAMISLAKKIDVQTGRHPYE |
| IEDB: Bepipred  (from structural protrusion) | MDGTLFPGDDDLAIPATEFFSTKAAKNPETKREAIVKAYGDDNEETLKITNITTKFEQIEKCCKRNDEVLFRLENHAETLRAAMISLAKKIDVQTGRHPYE |
| CBTOPE | MDGTLFPGDDDLAIPATEFFSTKAAKNPETKREAIVKAYGDDNEETLKITNITTKFEQIEKCCKRNDEVLFRLENHAETLRAAMISLAKKIDVQTGRHPYE |
| **A30L** | |
| Ellipro  (from protein sequence) | MNSLSIFFIVVATAAVCLLFIQSYSIYENYGNIKEFNATHAAFEYSKSIGGTPALDRRVQDVNDTISDVKQKWRCVVYPGNGFVSASIFGFQAEVGPNNTRSIRKFNTMRQCIDFTFSDVINIDIYNPCIAPNINNTECQFLKSVL |
| IEDB: Bepipred  (from structural protrusion) | MNSLSIFFIVVATAAVCLLFIQSYSIYENYGNIKEFNATHAAFEYSKSIGGTPALDRRVQDVNDTISDVKQKWRCVVYPGNGFVSASIFGFQAEVGPNNTRSIRKFNTMRQCIDFTFSDVINIDIYNPCIAPNINNTECQFLKSVL |
| CBTOPE | MNSLSIFFIVVATAAVCLLFIQSYSIYENYGNIKEFNATHAAFEYSKSIGGTPALDRRVQDVNDTISDVKQKWRCVVYPGNGFVSASIFGFQAEVGPNNTRSIRKFNTMRQCIDFTFSDVINIDIYNPCIAPNINNTECQFLKSVL |
| **A35R** | |
| Ellipro  (from protein sequence) | MMTPENDEEQTSVFSATVYGDKIQGKNKRKRVIGLCIRISMVISLLSMITMSAFLIVRLNQCMSANEAAITDSAVAVAAASSTHRKVASSTTQYDHKESCNGLYYQGSCYILHSDYKSFEDAKANCAAESSTLPNKSDVLTTWLIDYVEDTWGSDGNPITKTTSDYQDSDVSQEVRKYFCT |
| IEDB: Bepipred  (from structural protrusion) | MMTPENDEEQTSVFSATVYGDKIQGKNKRKRVIGLCIRISMVISLLSMITMSAFLIVRLNQCMSANEAAITDSAVAVAAASSTHRKVASSTTQYDHKESCNGLYYQGSCYILHSDYKSFEDAKANCAAESSTLPNKSDVLTTWLIDYVEDTWGSDGNPITKTTSDYQDSDVSQEVRKYFCT |
| CBTOPE | MMTPENDEEQTSVFSATVYGDKIQGKNKRKRVIGLCIRISMVISLLSMITMSAFLIVRLNQCMSANEAAITDSAVAVAAASSTHRKVASSTTQYDHKESCNGLYYQGSCYILHSDYKSFEDAKANCAAESSTLPNKSDVLTTWLIDYVEDTWGSDGNPITKTTSDYQDSDVSQEVRKYFCT |
| **L1R** | |
| Ellipro  (from protein sequence) | MDHNQYLLTMFFADDDSFFKYFASQDDESSLSDILQITQYLDFLLLLLIQSKNKLEAVGHCYESLSEEYRQLTKFTDSQDFKKLFNKVPIVTDGRVKLNKGYLFDFVISLMRFKKESALATTAIDPVRYIDPRRDIAFSNVMDILKSNKVEK |
| IEDB: Bepipred  (from structural protrusion) | MDHNQYLLTMFFADDDSFFKYFASQDDESSLSDILQITQYLDFLLLLLIQSKNKLEAVGHCYESLSEEYRQLTKFTDSQDFKKLFNKVPIVTDGRVKLNKGYLFDFVISLMRFKKESALATTAIDPVRYIDPRRDIAFSNVMDILKSNKVEK |
| CBTOPE | MDHNQYLLTMFFADDDSFFKYFASQDDESSLSDILQITQYLDFLLLLLIQSKNKLEAVGHCYESLSEEYRQLTKFTDSQDFKKLFNKVPIVTDGRVKLNKGYLFDFVISLMRFKKESALATTAIDPVRYIDPRRDIAFSNVMDILKSNKVEK |
| **M1R** | |
| Ellipro  (from protein sequence) | MGAAASIQTTVNTLSERISSKLEQEANASAQTKCDIEIGNFYIRQNHGCNITVKNMCSADADAQLDAVLSAATETYSGLTPEQKAYVPAMFTAALNIQTSVNTVVRDFENYVKQTCNSSAVVDNKLKIQNVIIDECYGAPGSPTNLEFINTGSSKGNCAIKALMQLTTKATTQIAPRQVAGTGVQFYMIVIGVIILAALFMYYAKRMLFTSTNDKIKLILANKENVHWTTYMDTFFRTSPMIIATTDIQN |
| IEDB: Bepipred  (from structural protrusion) | MGAAASIQTTVNTLSERISSKLEQEANASAQTKCDIEIGNFYIRQNHGCNITVKNMCSADADAQLDAVLSAATETYSGLTPEQKAYVPAMFTAALNIQTSVNTVVRDFENYVKQTCNSSAVVDNKLKIQNVIIDECYGAPGSPTNLEFINTGSSKGNCAIKALMQLTTKATTQIAPRQVAGTGVQFYMIVIGVIILAALFMYYAKRMLFTSTNDKIKLILANKENVHWTTYMDTFFRTSPMIIATTDIQN |
| CBTOPE | MGAAASIQTTVNTLSERISSKLEQEANASAQTKCDIEIGNFYIRQNHGCNITVKNMCSADADAQLDAVLSAATETYSGLTPEQKAYVPAMFTAALNIQTSVNTVVRDFENYVKQTCNSSAVVDNKLKIQNVIIDECYGAPGSPTNLEFINTGSSKGNCAIKALMQLTTKATTQIAPRQVAGTGVQFYMIVIGVIILAALFMYYAKRMLFTSTNDKIKLILANKENVHWTTYMDTFFRTSPMIIATTDIQN |
| **E8L** | |
| Ellipro  (from protein sequence) | MPQQLSPINIETKKAISDARLKTLDIHYNESKPTTIQNTGKLVRINFKGGYISGGFLPNEYVLSTIHIYWGKEDDYGSNHLIDVYKYSGEINLVHWNKKKYSSYEEAKKHDDGIIIIAIFLQVSDHKNVYFQKIVNQLDSIRSANMSAPFDSVFYLDNLLPSTLDYFTYLGTTINHSADAAWIIFPTPINIHSDQLSKFRTLLSSSNHEGKPHYITENYRNPYKLNDDTQVYYSGEIIRAATTSPVRENYFMKWLSDLREACFSYYQKYIEGNKTFAIIAIVFVFILTAILFLMSQRYSREKQN |
| IEDB: Bepipred  (from structural protrusion) | MPQQLSPINIETKKAISDARLKTLDIHYNESKPTTIQNTGKLVRINFKGGYISGGFLPNEYVLSTIHIYWGKEDDYGSNHLIDVYKYSGEINLVHWNKKKYSSYEEAKKHDDGIIIIAIFLQVSDHKNVYFQKIVNQLDSIRSANMSAPFDSVFYLDNLLPSTLDYFTYLGTTINHSADAAWIIFPTPINIHSDQLSKFRTLLSSSNHEGKPHYITENYRNPYKLNDDTQVYYSGEIIRAATTSPVRENYFMKWLSDLREACFSYYQKYIEGNKTFAIIAIVFVFILTAILFLMSQRYSREKQN |
| CBTOPE | MPQQLSPINIETKKAISDARLKTLDIHYNESKPTTIQNTGKLVRINFKGGYISGGFLPNEYVLSTIHIYWGKEDDYGSNHLIDVYKYSGEINLVHWNKKKYSSYEEAKKHDDGIIIIAIFLQVSDHKNVYFQKIVNQLDSIRSANMSAPFDSVFYLDNLLPSTLDYFTYLGTTINHSADAAWIIFPTPINIHSDQLSKFRTLLSSSNHEGKPHYITENYRNPYKLNDDTQVYYSGEIIRAATTSPVRENYFMKWLSDLREACFSYYQKYIEGNKTFAIIAIVFVFILTAILFLMSQRYSREKQN |

**Table S3.** **Molecular docking analysis:** The binding interaction between the vaccine and receptors.

| **System** | **Residue involved in hydrogen bonding** | | **Residues involved in hydrophobic interactions** | |
| --- | --- | --- | --- | --- |
|  | Vaccine (Chain B) | Receptor (Chain A) | Vaccine (Chain B) | Receptor (Chain A) |
| Vaccine + TLR1 | K10  T440  R473  S37  K431  G477  E14 | S454  N280  S219  Q479  H78  T198  K456 | N381, P398, W380, G399, P396, Q443, W7, F3, P458, G457, G455, P456, D460, K451, T401, W472, G475, G39, I38, T434, P432, T433, P476, P478, Y34 | R80, Y56, Q306, S477, T501, N252, N253, H453, S432, Q407, M381, N478, Q54, F123, K481, P503, K458, H102, H78, T198, Q173, V195, N220, F196, N199, I434 |
| Vaccine +TLR2 | T413, T412  H394  K370  K393  S386  Y362  E206  R253  S100  G98 | R447  M490, N470  N470  Q510  K512  N468  N61  S60  G41, S42  R63 | Y374, E390, Q65, K6, K369, W366, G71, I250, Y104, P241, G240, Y239, T248, T247, T101, P217 | K488, H318, K347, F322, H426, H449, H398, T84, D58, S39, N62, N44, D182, H206, M159, D231, K260, K208, H104, K37 |
| Vaccine + TLR4 | E189  E309  Y531  S37  A191  T233  E235 | H458  R460  G587  W550  Q507  D294  N265 | K188, F3, K287, K10, W7, I38, N462, G459, P458, Q534, G457, P234, Y239, K23, N21, G20, F332, K18, F334, L192, S190 | T553, E485, G579, Q578, H552, S580, K588, P589, K582, R591, K341, L293, F263, E266, M41, Q39, V32, H431, Q505, H456, G480 |
| Vaccine + TLR6 | H394  K370  E390  G216  K66  S100  R512  K121 | K377  S403  R378  Q224  Y501  R124  T197  N144 | T413, Y362, K393, P217, Y365, I75, E72, G71, A251, K369, P97, Q254, R253, P252, E124, G214, P215, F219, K410, G127, G125 | K453, L431, S404, C173, H200, R531, G528, K451, Q500, H125, E428, K147, H170, L148, T146, S172, T149, K140, E141, H222, Y284, Q257, Q380, A121 |
| Vaccine + MHC-I | K410  G216  Y362  P252  Q249  G218  T247  S373 | H188  Y27  H191  R21  T94  Q96  D119  E275K | K393, P215, P217, P411, T413, A251, T248, F219, W366, K370, K369 | W204, T190, V125, I23, R202, H192, T10, A193, S92, V12, F8, W274 |
| Vaccine + MHC-II * | Q254  K279  R512  R513  D148  V178  V255  G257  E177  Q520 | K39 (a)  K75 (a)  E55 (a)  E55 (a)  K67 (a)  D17 (a)  Q57 (a)  Q57 (a)  R4 (b)  T77 (b) | Y120, Q 176, S282, Y280, S281, E177, R179, K180, P304, A256, P260, Y306, S122  D514, E284, Y283, N299, P168, E301 | E40 (a), E71 (a), Y79 (a), T74 (a), V116 (a), F54 (a), S53 (a), A68 (a), I72 (a), R76 (a), Q18 (a), V65 (a), A64 (a), A37 (a)  H81 (b), Y60 (b), Q64 (b), G1 (b), L67 (b), D66 (b), Q70 (b) |

* The two chains of MHC-II is denoted by chain A = (a); chain B= (b)
